# Mucosal Immunization with DTaP Confers Protection against *Bordetella pertussis* Infection and Cough in Sprague-Dawley Rats

**DOI:** 10.1128/IAI.00346-21

**Published:** 2021-11-16

**Authors:** Jesse M. Hall, Graham J. Bitzer, Megan A. DeJong, Jason Kang, Ting Y. Wong, M. Allison Wolf, Justin R. Bevere, Mariette Barbier, F. Heath Damron

**Affiliations:** a Department of Microbiology, Immunology and Cell Biology, School of Medicine, West Virginia University, Morgantown, West Virginia, USA; b Vaccine Development Center, West Virginia University Health Sciences Center, Morgantown, West Virginia, USA; Washington State University

**Keywords:** *Bordetella pertussis*, DTaP, immunization, mucosal, pertussis, plethysmography, rats

## Abstract

Pertussis is a respiratory disease caused by the Gram-negative pathogen, Bordetella pertussis. The transition from a whole-cell pertussis vaccine (wP and DTP) to an acellular pertussis vaccine (aP, DTaP, and Tdap) correlates with an increase in pertussis cases, despite widespread vaccine implementation and coverage, and it is now appreciated that the protection provided by aP rapidly wanes. To recapitulate the localized immunity observed from natural infection, mucosal vaccination with aP was explored using the coughing rat model of pertussis. Overall, our goal was to evaluate the route of vaccination in the coughing rat model of pertussis. Immunity induced by both oral gavage and intranasal vaccination of aP in *B. pertussis* challenged rats over a 9-day infection was compared to intramuscular wP (IM-wP)- and IM-aP-immunized rats that were used as positive controls. Our data demonstrate that mucosal immunization of aP resulted in the production of anti-*B. pertussis* IgG antibody titers similar to IM-wP- and IM-aP-vaccinated controls postchallenge. IN-aP also induced anti-*B. pertussis* IgA antibodies in the nasal cavity. Immunization with IM-wP, IM-aP, IN-aP, and OG-aP immunization protected against *B. pertussis*-induced cough, whereas OG-aP immunization did not protect against respiratory distress. Mucosal immunization by both intranasal and oral gavage administration protected against acute inflammation and decreased bacterial burden in the lung compared to mock-vaccinated challenge rats. The data presented in this study suggest that mucosal vaccination with aP can induce a mucosal immune response and provide protection against *B. pertussis* challenge. This study highlights the potential benefits and uses of the coughing rat model of pertussis; however, further questions regarding waning immunity still require additional investigation.

## INTRODUCTION

Infection of the respiratory mucosa by the Gram-negative bacterium Bordetella pertussis (*B. pertussis*) causes the disease known as pertussis (whooping cough) (1). Clinical manifestations of pertussis are characterized by paroxysmal cough, hypertension, leukocytosis, and—in severe cases—death, particularly in infants who have yet to receive their first vaccine dose (2–4). Before pertussis vaccines were introduced in the United States, pertussis led to approximately 200,000 deaths annually (5). Largely, this disease has been under control by the use of diphtheria tetanus whole-cell pertussis (DTP and wP) and acellular pertussis (DTaP, Tdap, and aP) vaccines. DTP was introduced in the 1940s and 1950s and was largely effective in decreasing pertussis incidence (6). Due to the robust immune response and reactogenicity concerns, developed countries converted to the use of DTaP in the 1990s (2).

Since the introduction of the aP vaccine, pertussis cases have been increasing, despite high vaccine coverage. It has been hypothesized that the increase in pertussis cases can be attributed to waning immunity from DTaP and Tdap vaccination, vaccine-driven evolution of *B. pertussis* strains, increased surveillance of pertussis, increased asymptomatic transmission, and improved PCR-based molecular identification of cases (7–13). Infant baboons vaccinated with DTaP (one human dose administered at 2, 4, and 6 months of age) were still colonized after experimental *B. pertussis* challenge in addition to subsequent transmission to naive baboons (14). In the same study, Warfel et al. showed that convalescent baboons that cleared a prior *B. pertussis* infection were not colonized after rechallenge of *B. pertussis* 1 month later (14). In humans, studies suggest that convalescent immunity can confer protection for approximately 4 to 20 years (15), while DTaP immunity falls short, lasting on average 4 to 12 years (15). Overall, these data demonstrated that immunity induced through natural infection can generate longer-lasting protection that also elicits pathogen clearance. Since *B. pertussis* is a respiratory pathogen, it can be hypothesized by many in the field that generating an immune response at the respiratory mucosa is necessary for protection against pertussis.

Infection with *B. pertussis* localized to respiratory epithelium primes the immune response against subsequent *B. pertussis* infection by recruiting antibody-producing cells and tissue-resident memory T cells (Trms) (16). Bacteria invading mucosal surfaces can induce inflammation, resulting in the subsequent production of IgA antibodies (17). Patients previously infected with *B. pertussis* have developed IgA antibody titers in their nasal secretions (18). In addition, anti-*B. pertussis* IgA antibodies from patients who have convalesced from *B. pertussis* infection inhibit bacterial attachment *in vitro* and increase *B. pertussis* uptake and killing by human polymorphonuclear leukocytes (19, 20). Convalescent humans also generate *B. pertussis*-specific IgG antibody titers that correlate with protection (21, 22). *B. pertussis*-infected mice generate *B. pertussis*-specific CD4^+^ T cells in the lung that secrete gamma interferon and interleukin-17 (IL-17) (23, 24). In mice, *B. pertussis* infection induced Trms in the lung and were associated with pathogen clearance (16). Nonhuman primates previously infected with *B. pertussis* generate both Th1 and Th17 memory cells that are still detected 2 years postinfection (25). To induce a similar protective immune response that is observed during natural infection, mucosal vaccination has recently been investigated by our laboratory and others (26–28).

Mucosal immunization has been rarely used as a vaccination strategy to generate a protective immune response against pertussis in clinical and preclinical models. Oral vaccination with wP induced the production of *B. pertussis*-specific antibody titers in both the saliva and the sera of newborns (29). In a subsequent study in 1985, oral immunization with 10^12^ CFU of killed *B. pertussis* led to the induction of antibody titers in the saliva and sera of newborns (30). The frequency of pertussis was lower in orally vaccinated newborns during the first year of life compared to newborns who were unvaccinated, although this difference disappeared by the end of the year (30). In mice, oral administration of the attenuated bacterial vectors Salmonella Typhimurium and Escherichia coli expressing the *B. pertussis* antigen, filamentous hemagglutinin (FHA), results in the production of anti-FHA IgA antibody titers in the lung (31). Intranasal (i.n.) immunization of a live attenuated vaccine strain of *B. pertussis*, BPZE1, was protective both in preclinical and clinical studies (32–35). Previously, our lab has shown that i.n. immunization of DTaP can induce a protective immune response in mice (27, 28). Boehm et al. illustrated that i.n. vaccination of DTaP with or without the addition of the adjuvant curdlan induces both anti-*B. pertussis* and anti-pertussis toxin (anti-PT) IgG antibody titers, as well as *B. pertussis*-specific IgA in the lung (27). A follow-up study performed by Wolf et al. suggested that i.n. aP (IN-aP) vaccination is protective through the induction of humoral responses 6 months after booster vaccination and challenge (28). Mounting evidence supports that mucosal immunization can be protective against *B. pertussis* colonization, but unfortunately murine studies lack the ability to evaluate one the of hallmark symptoms of pertussis, *B. pertussis*-induced coughing.

The rat model of pertussis has been utilized to characterize *B. pertussis* pathogenesis and evaluate coughing manifestation from *B. pertussis* infection (36–42). Only a few studies have been performed investigating vaccine efficacy in the coughing rat model of pertussis. Hall et al. demonstrated that the SmithKline Beecham three-component aP vaccine (detoxified PT [PTd], FHA, and a 69-kDa antigen, presumably pertactin [PRN]), the Connaught five-component vaccine (PTd, FHA, agglutinins 2+3 [fimbriae], and PRN), and Evans whole-cell pertussis vaccine protected against cough upon intrabronchial *B. pertussis* challenge (41). Rats administered a single human dose of DTP had a lower incidence of coughing following *B. pertussis* challenge (39). In the present study, our overarching goal was to evaluate various routes of immunization of DTaP in the coughing rat model of pertussis. We hypothesized that both oral and i.n. vaccination would protect against bacterial burden upon challenge, as well as against *B. pertussis*-induced coughing, in the rat model of pertussis by generating a protective immune response at the site of infection. To test this hypothesis, we i.n. and oral gavage (o.g.) vaccinated and challenged Sprague-Dawley rats with a 1/5 human dose of DTaP. Intramuscular wP (IM-wP)- and IM-aP-vaccinated and *B. pertussis*-challenged rats were used as positive controls to compare vaccine-mediated immunity since intramuscular (i.m.) administration of DTaP is the current route of vaccine administration.

In our study, we aimed to use the rat model of pertussis to measure protection induced from mucosal vaccination of DTaP. By utilizing the coughing rat model of pertussis, protection against bacterial burden in the respiratory tract and prevention of *B. pertussis* induced cough after immunization was critically analyzed. We also focused on evaluating the serological responses regarding vaccination followed by *B. pertussis* challenge. Our data support the hypothesis that protection is afforded by mucosal immunization with DTaP. We found that i.n. and o.g. immunization with DTaP not only induced systemic anti-*B. pertussis* IgG antibodies but also induced mucosal anti-*B. pertussis* IgA antibodies. These data suggest that oral vaccination of DTaP can generate a humoral immune response at the respiratory mucosa in rats. IN-aP and o.g. AP (OG-aP) was also capable of protecting against *B. pertussis-*induced cough. Furthermore, mucosal vaccination protected against bacterial burden in the respiratory tract. In conclusion, we highlight here (i) the benefits of using the coughing rat model of pertussis and (ii) the prospect of mucosal vaccination with *B. pertussis* vaccines.

## RESULTS

### Intranasal immunization induces systemic anti-*B. pertussis* IgG and anti-PT IgM and IgG antibody titers following booster immunization.

**FIG 1:**
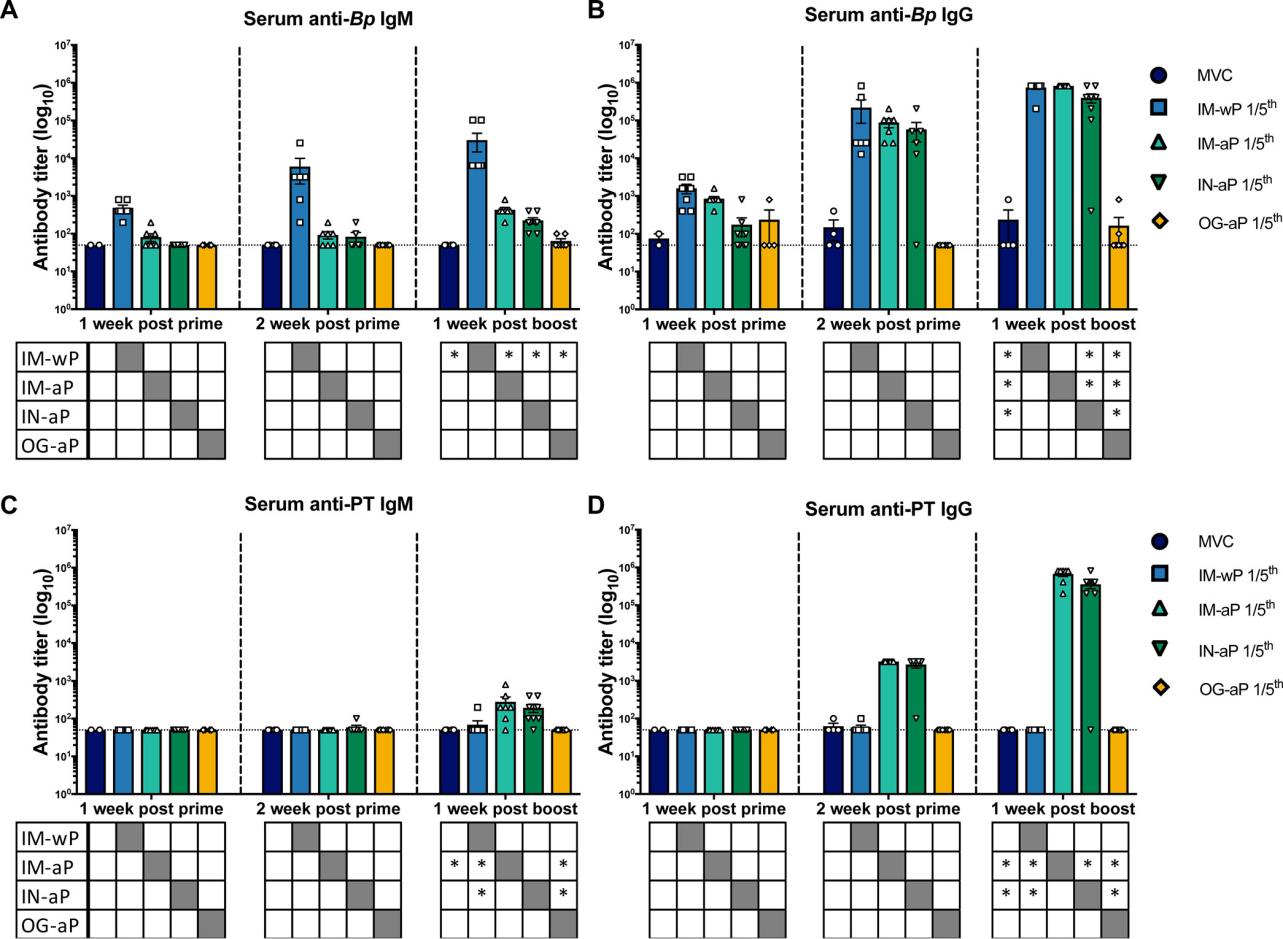
i.n. booster vaccination induces systemic anti-*B. pertussis* (anti-*Bp*) and anti-PT antibody titers. At 1 and 2 weeks after prime immunization and at 1 week after boost blood was collected via the saphenous vein, and anti-*Bp* and anti PT IgM (A and C)- and IgG (B and D)-specific antibodies were measured. The results are shown on a log scale and as means ± the standard errors of the mean (SEM; *n* = 3 to 8). The dotted line represents the limit of detection. *, *P* < 0.05. (*n* = 4 to 8). *P* values were determined by two-way ANOVA with Tukey’s *post hoc* test compared between groups. Asterisks (*) under each graph annotate the significance between labeled groups under the *y* axis and the group under the corresponding bar. Grayed-out boxes indicate that no stats were calculated.

We hypothesized that IN-aP and OG-aP immunization would induce systemic IgM and IgG antibodies, since mucosal immunization stimulates the induction of both systemic and mucosal antibodies (27, 28, 30). To test this hypothesis, 3-week-old Sprague-Dawley rats were IM-wP, IM-aP, IN-aP, and OG-aP immunized, followed by a booster immunization at 6 weeks of age with the same corresponding vaccine (see Fig. S1). Mucosal and systemic antibodies were measured over the course of vaccination (see Fig. S1). Minimal differences in antibody titers prior to booster vaccination were observed for all immunized groups (Fig. 1). Compared to all other groups, IM-wP vaccination induced a significant increase of anti-*B. pertussis* IgM in the serum 1 week after booster vaccination (Fig. 1A). IM-wP-, IM-aP-, and IN-aP-vaccinated rats had a significant increase in anti-*B. pertussis* IgG antibody titers following booster immunization compared to mock-vaccinated challenged (MVC) rats (Fig. 1B). Rats vaccinated using IM-aP experienced a significant increase in anti-PT IgM antibody titers 1 week after the booster vaccine compared to the MVC control (Fig. 1C). IM-aP- and IN-aP-vaccinated rats had a significant increase in anti-PT IgM antibodies compared to IM-wP- and OG-aP-vaccinated rats (Fig. 1C). This same trend was also observed in measuring anti-PT IgG antibodies (Fig. 1D). We observed a significant 4-log increase in anti-PT IgG antibodies in IM-aP- and IN-aP-immunized rats compared to our MVC, IM-wP, and OG-aP groups (Fig. 1D). Here, our data show that after booster immunization, IN-aP-vaccinated rats developed systemic anti-*B. pertussis* IgG and anti-PT IgG and IgM antibody titers.

### Mucosal vaccination protects against cough from *B. pertussis*-infected rats.

**FIG 2:**
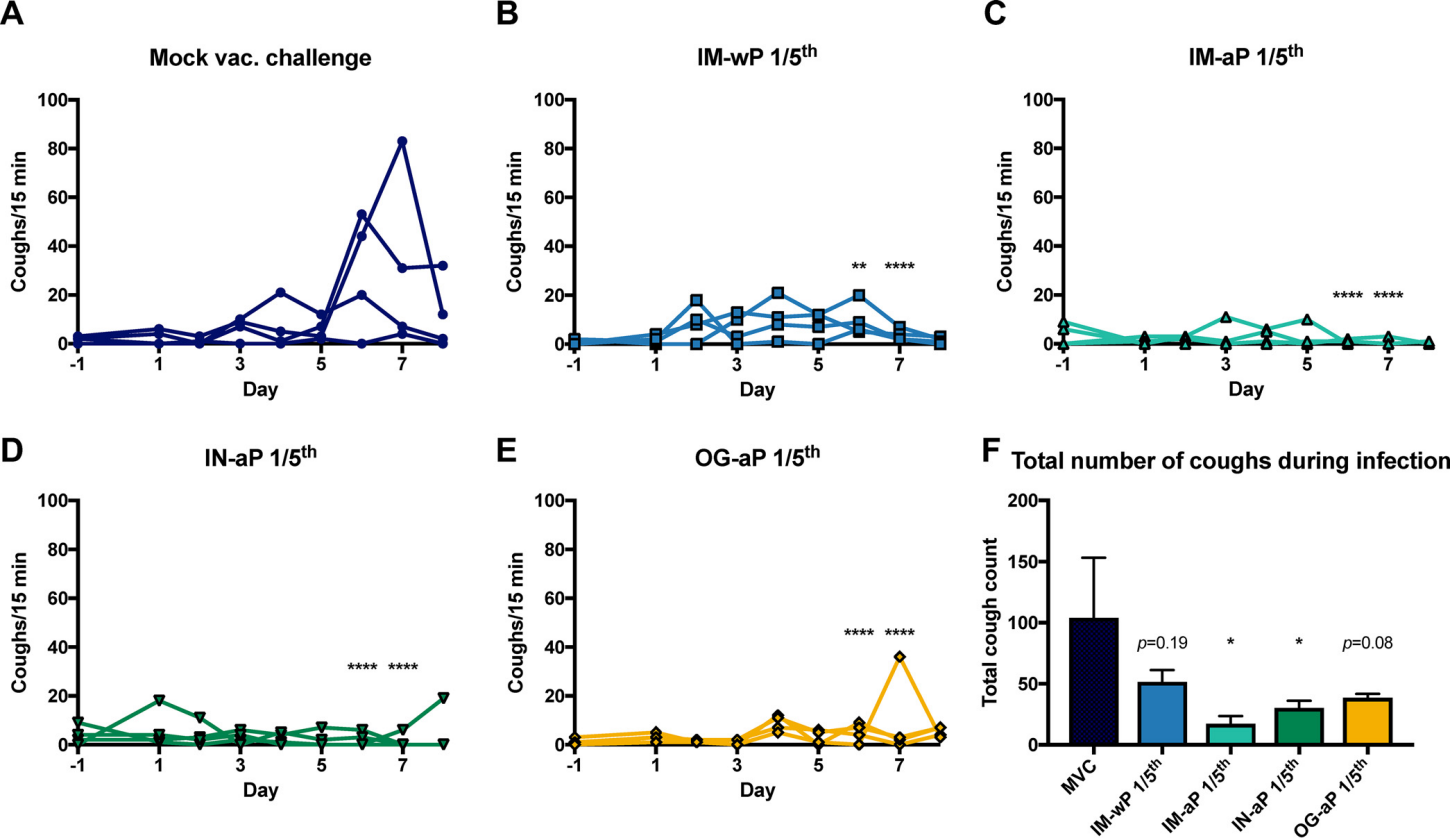
Intranasal and oral vaccination of acellular pertussis vaccine decreases cough in B. pertussis-infected rats. Coughs were counted every day of the 9-day infection using WBP. Coughs were counted for mock-vaccinated challenge rats (A) and for IM-wP (B)-, IM-aP (C)-, IN-aP (D)-, and OG-aP (E)-vaccinated and challenged rats. (F) To assess any potential differences between vaccine groups over the entire course of infection, the average total numbers of coughs for each rat per group were compared. The results are shown as means ± the SEM (*n *= 3 to 4). *P* values were determined by two-way ANOVA with Dunnett’s *post hoc* test and one-way ANOVA with Dunnett *post hoc* test for total cough count (*, *P *<* *0.05; **, *P *<* *0.01; ***, *P *<* *0.001; ****, *P *<* *0.0001 [compared to the mock-vaccinated challenge control group]).

In 2014, Warfel et al. showed that aP vaccination was protective against pertussis disease but failed to protect against colonization and transmission of *B. pertussis* in the nonhuman primate model (14). We hypothesized that mucosal vaccination with DTaP would protect against *B. pertussis*-induced cough by eliciting a protective immune response at the respiratory mucosa. To test this hypothesis, vaccinated rats were subsequently i.n. challenged with *B. pertussis* 2-weeks after booster vaccine administration (see Fig. S1). Every evening postchallenge, coughs were counted using whole-body plethysmography (WBP). MVC rats averaged a total of five coughs or less during monitoring for the first 5 days of infection (Fig. 2A). At days 6 and 7 postchallenge, average coughs per 15 min increased to more than 30 coughs (Fig. 2A). There was a significant decrease in coughs for rats vaccinated with IM-wP, IM-aP, IN-aP, and OG-aP at days 6 and 7 postchallenge compared to MVC rats (Fig. 2B to E). On average, rats vaccinated with IM-wP coughed ∼6 coughs per 15 min each day (Fig. 2B). Rats vaccinated with IM-aP on average coughed twice per 15 min each day (Fig. 2C). IN-aP-immunized rats on average coughed three times per 15 min each day, while OG-aP-vaccinated rats coughed on average four times per 15 min each day (Fig. 2D and E). To compare the average total number of coughs each day postchallenge, we calculated the total number of coughs for each group per animal. We observed a significant decrease in total number of coughs in rats vaccinated with IM-aP (14 coughs) and IN-aP (27 coughs) compared to MVC rats (102 coughs) over the 9-day infection (Fig. 2F). OG-aP-immunized rats coughed, on average, 35 times (Fig. 2F). Our data demonstrate that mucosal vaccination in rats protects against *B. pertussis*-induced cough.

### Intranasal vaccination protects against pulmonary distress.

**FIG 3:**
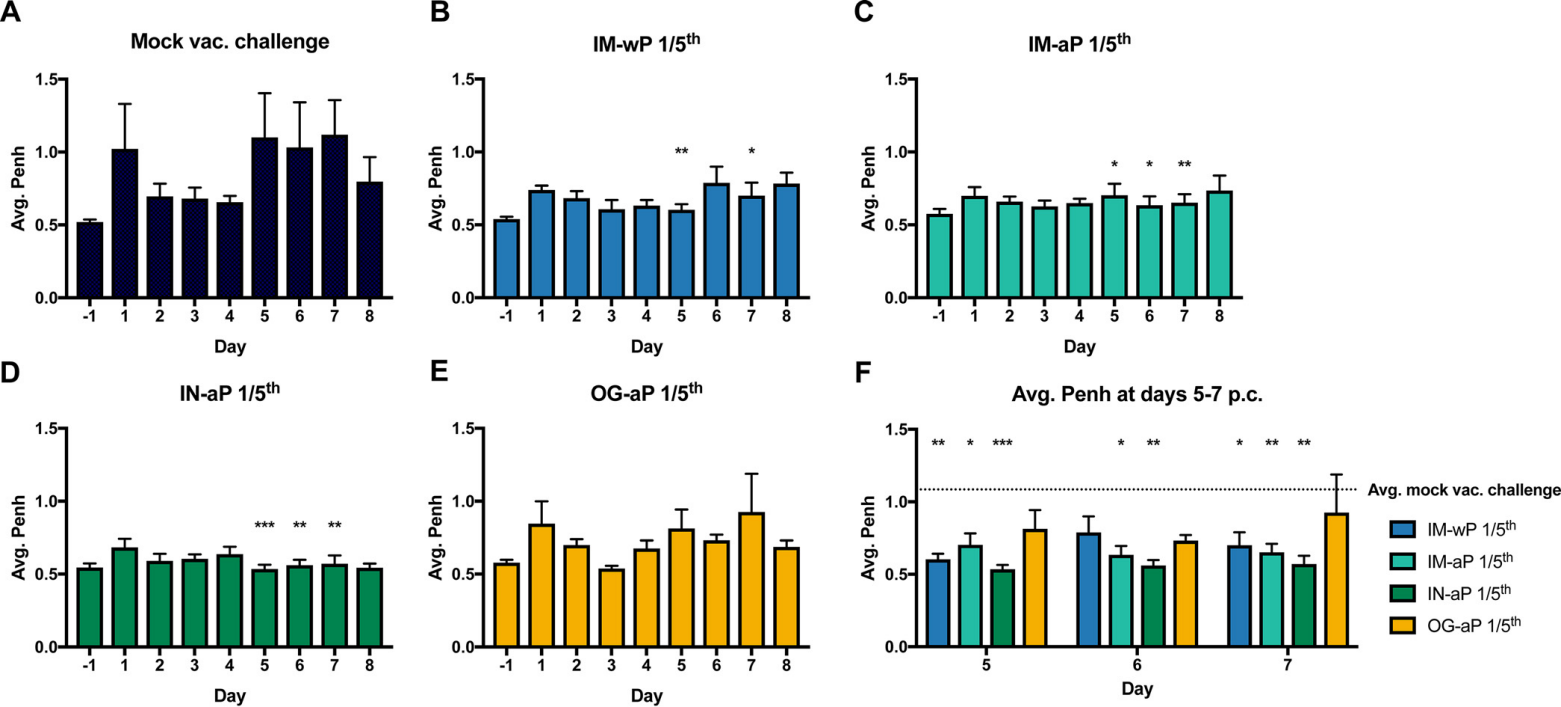
Intranasal vaccination decreases the pulmonary restriction of B. pertussis-infected rats. Bronchiole restriction was measured over the course of infection by WBP. Bronchiole restriction was determined by the factor Penh for mock-vaccinated challenge rats (A) and for IM-wP (B)-, IM-aP (C)-, IN-aP (D)-, and OG-aP (E)-vaccinated and challenged rats. The results are shown as means ± the SEM (*n *=* *3 to 4). (F) Differences observed between groups at days 5 to 7 postchallenge. *P* values were determined by two-way ANOVA with Dunnett’s *post hoc* test (*, *P *<* *0.05; **, *P *<* *0.01; ***, *P* < 0.001 [compared to the mock-vaccinated challenge group]).

Our previous work has shown that *B. pertussis*-infected rats showed a significant increase in pulmonary distress after challenge (43). Pulmonary distress can be evaluated by calculating the enhanced pause (PenH). PenH functions as a representation of bronchoconstriction, taking into consideration the timing between early and late expiration and the estimated maximum inspiratory and expiratory flows per breath. We hypothesized that mucosal vaccination would protect against *B. pertussis*-induced pulmonary distress. Here, rats vaccinated with IM-wP, IM-aP, and IN-aP had a significant decrease in PenH compared to the MVC control group at days 5 and 7 postchallenge (Fig. 3A, D, E, and F). Rats vaccinated IM-aP and IN-aP also experienced a significant decrease in PenH at day 6 postchallenge compared to MVC rats (Fig. 3A, C, D, and F). However, there was no significant decrease in PenH in OG-aP-vaccinated rats compared to MVC, suggesting that the induced immune response is not sufficient enough to protect rats from *B. pertussis*-induced respiratory distress (Fig. 3E). Other respiratory parameters were also measured using WBP (see Fig. S2). In brief, we observed that rats vaccinated with IM-wP, IM-aP, and IN-aP had a significant decrease in pause (PAU) compared to the MVC control group, which is another indicator of bronchiole restriction (see Fig. S2B). Rats vaccinated with the aP vaccine regardless of route also showed a significant decrease in tidal volume (TVb) compared to MVC rats, which could be crudely associated with inflammation (see Fig. S2D). Overall, our data demonstrated that IN-aP vaccination decreases pulmonary distress of *B. pertussis*-infected rats.

### Mucosal immunization induces production of *B. pertussis*-specific antibodies in serum, while i.n. immunization also induces PT-specific antibodies in serum.

**FIG 4:**
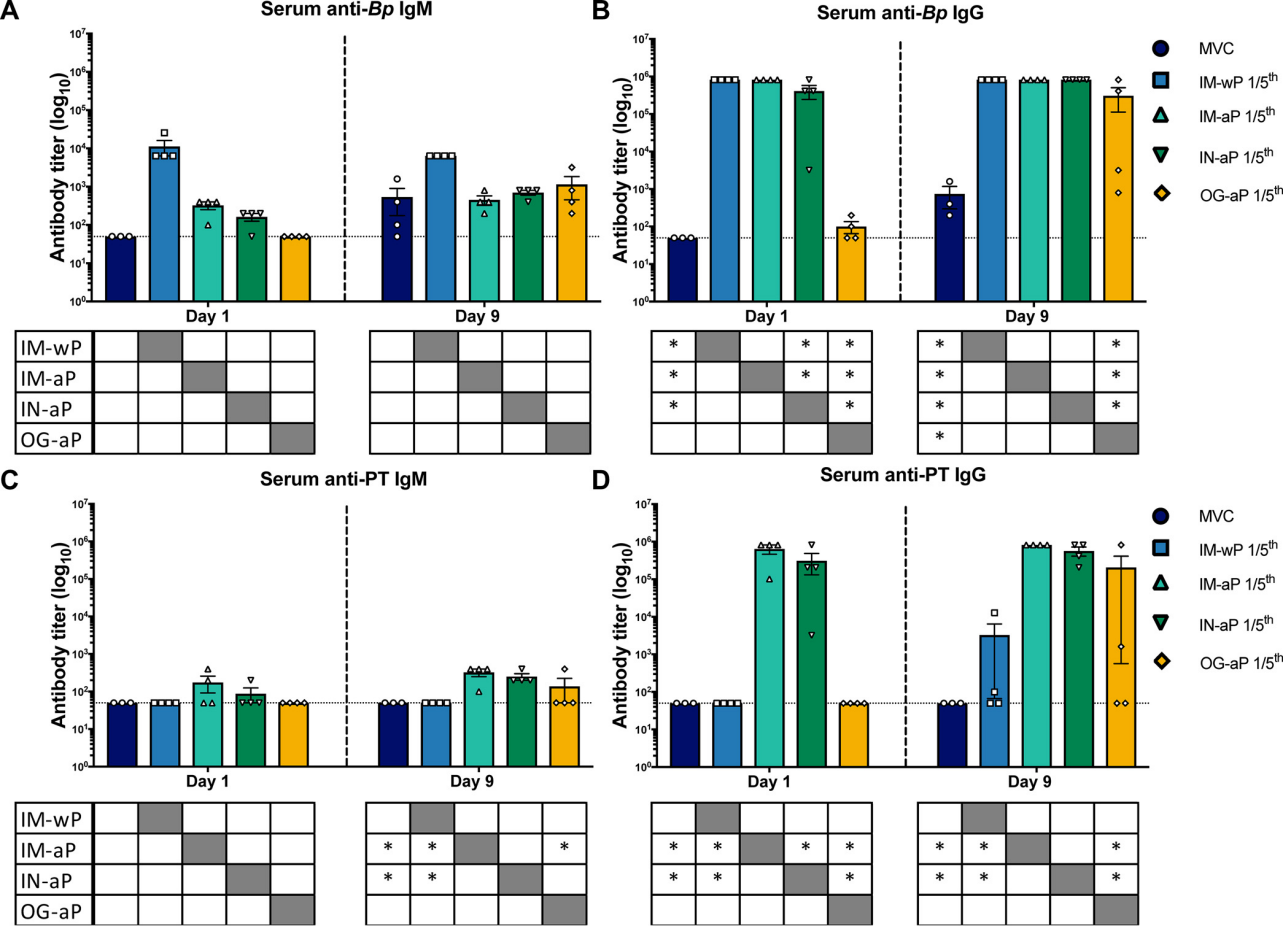
Mucosal vaccination induces the production of anti-*B. pertussis* IgG, whereas i.n. immunization also induces both anti-PT IgM and IgG antibodies. ELISAs were used to determine and compare the induced serological responses from vaccinated and challenge rats in the serum. Both IgM (A and C) and IgG (B and D) serum antibody titers from immunized and challenged rats were measured postchallenge. The dotted line represents the limit of detection. The results are shown on a log scale as means ± the SEM (*, *P* < 0.05; *n *=* *4). *P* values were determined by two-way ANOVA with Tukey’s *post hoc* test compared between groups. Asterisks (*) under each graph annotate the significance between labeled group under the *y* axis and the group under the corresponding bar. Grayed-out boxes indicate that no stats were calculated.

Next, we wanted to measure systemic antibody responses to *B. pertussis* and PT after challenge. IM-wP-vaccinated rats had a slight increase in anti-*B. pertussis* IgM antibodies compared to all other vaccinated groups, albeit not significant (Fig. 4A). We observed a significant increase in anti-*B. pertussis* IgG antibody titers in IM-wP-, IM-aP-, and IN-aP-vaccinated rats compared to the MVC at day 1 postchallenge (Fig. 4B). At day 9 postchallenge, all vaccinated rats had a significant increase of anti-*B. pertussis* IgG antibody titers compared to the MVC control (Fig. 4B). After *B. pertussis* challenge, IM-aP- and IN-aP-immunized rats had a significant increase in anti-PT IgM antibodies compared to IM-wP-immunized rats and MVC controls. (Fig. 4C). Similar results were observed in measuring anti-PT IgG titers (Fig. 4D). IM-aP and IN-aP vaccination induced a significant increase in anti-PT IgG antibody titers compared to MVC-, IM-wP-, and OG-aP-immunized rats after booster vaccination and at days 1 and 9 postchallenge (Fig. 4D). Although not significant, two of the OG-aP-immunized rats had detectable anti-PT IgG antibody titers in the serum at day 9 postchallenge (Fig. 4D). An enzyme-linked immune absorbent spot (ELISpot) assay was used to determine the number of *B. pertussis*-specific IgG cells in the bone marrow at day 9 postchallenge. There was an increase in the number of *B. pertussis*-specific IgG cells in the bone marrow in all vaccination groups compared to the MVC control; however, the only significant increase in *B. pertussis*-specific IgG-producing cells in the bone marrow was detected in IM-wP-vaccinated rats (see Fig. S3). Our data indicate that mucosal vaccination via i.n. and o.g. immunization induced *B. pertussis*-specific IgG antibody responses.

### Intranasal immunization induces production of *B. pertussis*-specific IgA antibodies in the nasal cavity.

**FIG 5:**
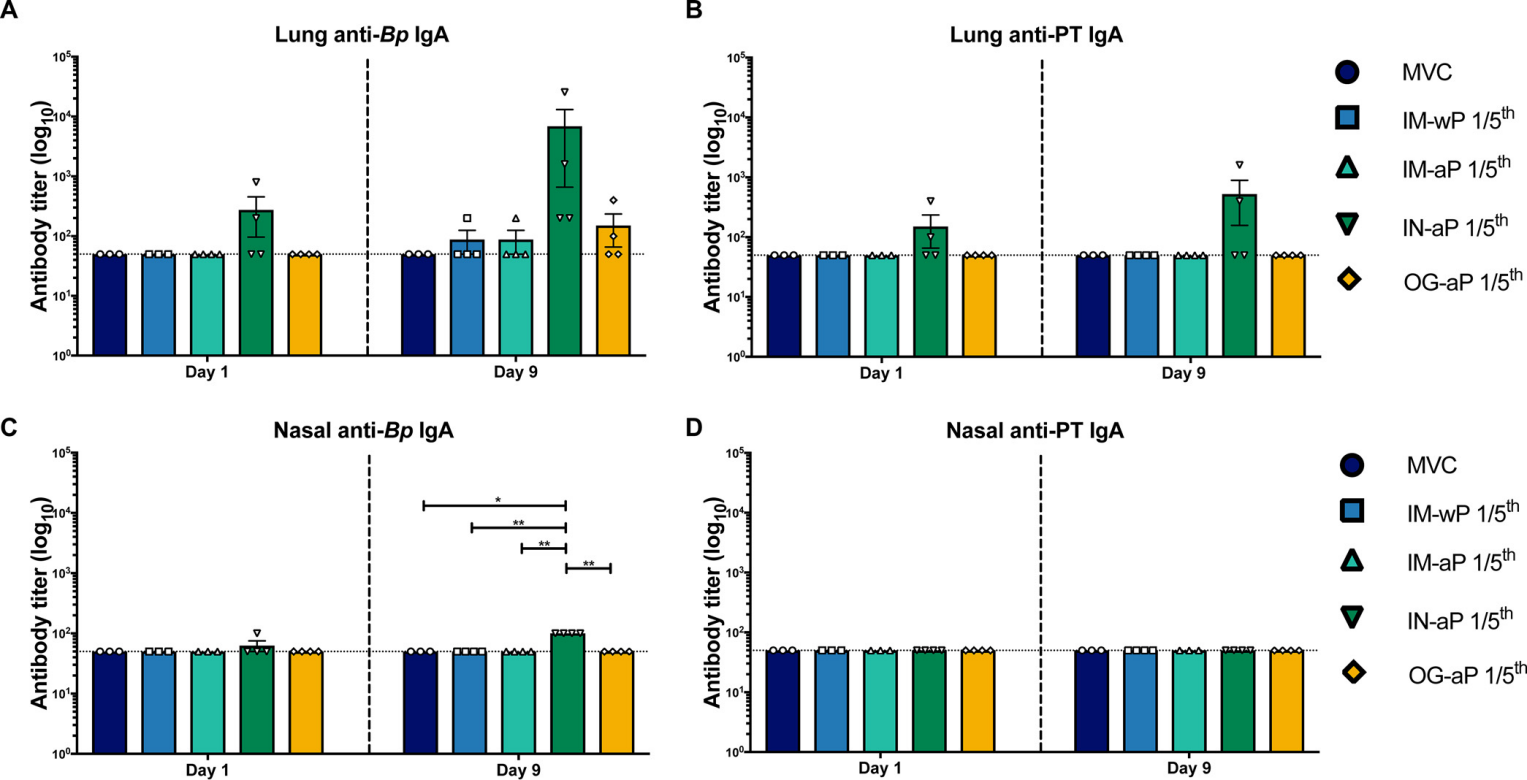
Intranasal immunization elicits the production of anti-*B. pertussis* IgA in the respiratory tract. ELISA was used to analyze antibodies in the lung (A and B) and nasal cavity (C and D) from lung homogenate supernatant and PBS flushed through the nasal cavity from vaccinated and challenge rats at days 1 and 9 postchallenge. IgA titers were determined against pertussis toxin and B. pertussis. The dotted line represents the limit of detection. The results are shown on a log scale as a means ± the SEM (*n* = 3 to 4; **, *P* < 0.01; ****, *P* < 0.0001). *P* values were determined by the Kruskal-Wallis test, using Dunn’s *post hoc* test to compare between groups.

In humans, previous *B. pertussis* infection leads to anti-*B. pertussis* IgA antibodies in nasal secretions (18). IgA antibodies to *B. pertussis* play a role in the inhibition of *B. pertussis* attachment *in vitro* to epithelial cells (19). Here, we investigated whether i.n. and o.g. immunization of DTaP would induce mucosal IgA antibodies in the lung and/or the nasal cavity. In the lung, three of the four IN-aP-vaccinated rats had detectable anti-*B. pertussis* IgA antibodies at day 1 postchallenge, although this was not significant (Fig. 5A). We did not detect anti-*B. pertussis* IgA antibody titers at day 1 postchallenge in the lungs of IM-wP-, IM-aP-, or OG-aP-vaccinated rats (Fig. 5A). It is possible that we needed to use a larger amount of serum or enhanced secondary detection methods to detect IgA at day 1. Low levels of anti-*B. pertussis* IgA titers were measured in all vaccinated groups at day 9 postchallenge, although this was not significant compared to the MVC control (Fig. 5A). The same trend was observed in the lung when we measured anti-PT IgA titers at day 1 postchallenge (Fig. 5B). At day 9 postchallenge, 50% of the IN-aP-vaccinated rats and 25% of the OG-aP-vaccinated rats had detectable anti-PT IgA (Fig. 5B). In the nasal cavity, only one IN-aP-vaccinated rat had detectable anti-*B. pertussis* IgA antibody titers; however, we did measure a significant increase in anti-*B. pertussis* IgA antibodies in IN-aP-immunized rats at day 9 postchallenge compared to the MVC-, IM-wP-, IM-aP-, and OG-aP-immunized rats (Fig. 5C). Only one IN-aP- and one OG-aP-vaccinated rat had detectable amounts of anti-PT IgA in the nasal cavity at day 9 postchallenge (Fig. 5D). Overall, our data reveal that IN-aP immunization can induce IgA antibodies in the nasal cavities of rats.

### Mucosal immunization protects against acute inflammation in the lung.

**FIG 6:**
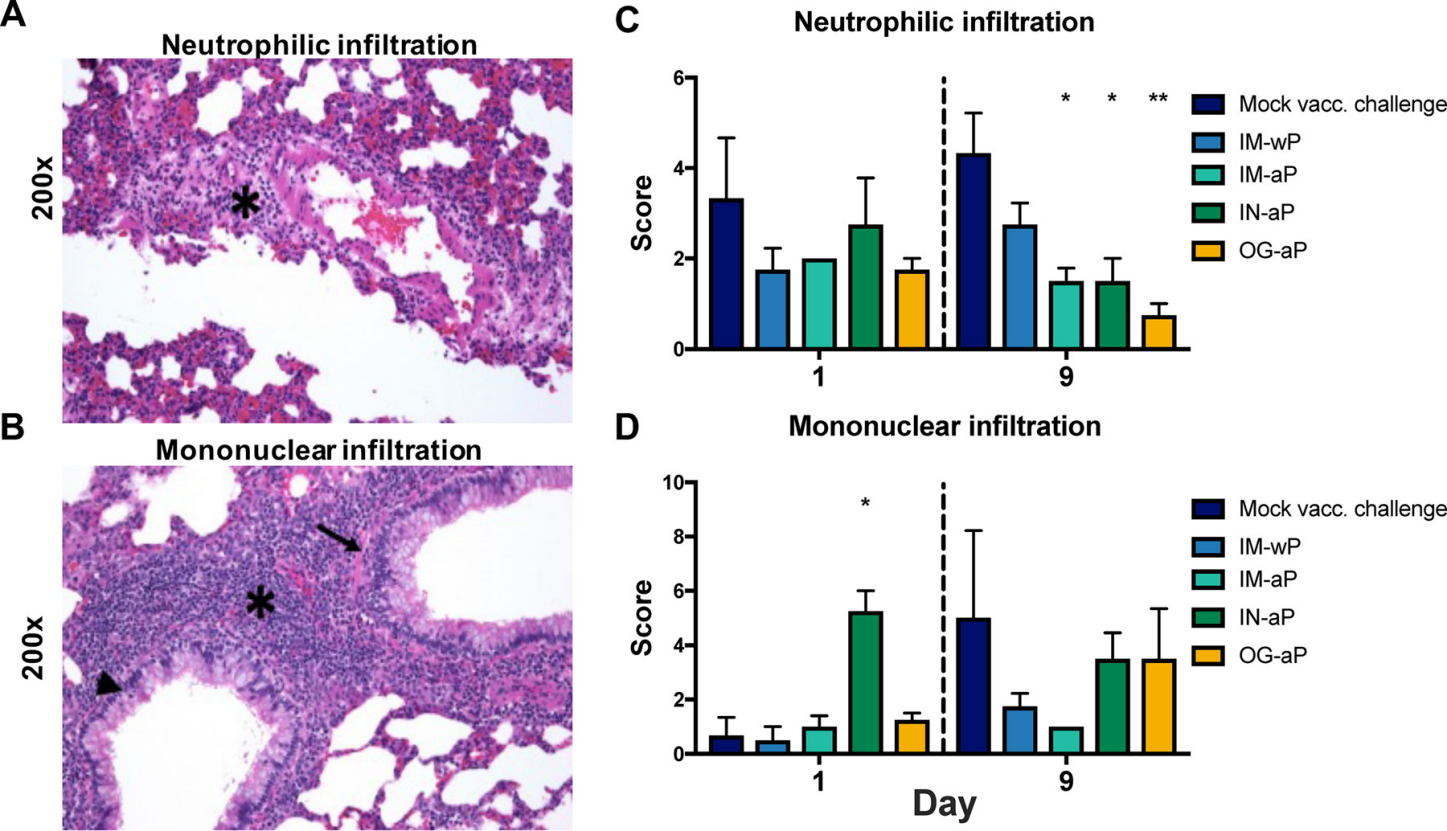
Mucosal vaccination protects against acute and total inflammation in the lung of *B. pertussis*-infected Sprague-Dawley rats. After euthanasia, the left lobe of the lung was excised, sectioned, and stained with H&E. Lung samples scores are based on standard qualitative toxicologic scoring criteria: 0, none; 1, minimal (rare); 2, mild (slight); 3, moderate; 4, marked; and 5, severe. (A) Representative image of acute inflammation of rat lung showing increased numbers of neutrophils and edema surrounding blood vessel (asterisk). (B) Representative image of chronic inflammation of the rat lung showing increased numbers of mononuclear cells surrounding bronchioles (asterisk). Inflammatory cells are also present in the lamina propria (arrow) and epithelium (arrowhead) of bronchioles. (C) Average acute inflammation scores of the lung are detailed by the presence of neutrophils in the parenchyma, blood vessels, and the airways. (D) Average chronic inflammation scores are distinguished by mononuclear infiltrates in the parenchyma, blood vessels, and airway of the lung. All scoring assessments were determined with no knowledge of the groups. The results are shown as means ± the SEM (*n *= 3 to 4). *P* values were determined by two-way ANOVA, followed by Dunnett’s comparison test (*, *P *<* *0.05; **, *P *<* *0.01 [compared to mock challenge]).

Our previous rat challenge study illustrated that i.n. *B. pertussis* challenge gave rise to both neutrophilic and mononuclear infiltration into the rat lung (43). Here, we used histology to assess whether mucosal immunization would protect against *B. pertussis*-induced inflammation in the lung. At day 1 postchallenge, no differences in neutrophilic inflammation were observed; however, at day 9 postchallenge, rats vaccinated with IM-aP, IN-aP, and OG-aP had significantly lower neutrophilic inflammation scores compared to the MVC rats (Fig. 6A and C). IN-aP-immunized rats showed an increase in mononuclear infiltration score at day 1 postchallenge (Fig. 6B and D). There were no observed differences in mononuclear infiltration score in vaccinated rats compared to MVC rats at day 9 postchallenge (Fig. 6D). There were no differences in lung weight, which can be used as a crude measurement for lung inflammation, following *B. pertussis* challenge (see Fig. S4A). We did observe a significant increase in percent body weight change in IM-wP-vaccinated rats compared to MVC control rats, suggesting that wP protects against the weight loss observed in nonvaccinated challenged rats (see Fig. S4B). Our observations suggest that that mucosal vaccination protects against *B. pertussis*-induced inflammation in the lung (Fig. 6C).

### Mucosal vaccination protects against *B. pertussis* challenge.

**FIG 7:**
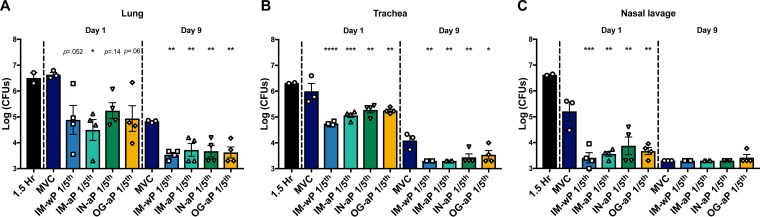
Oral and intranasal immunization decreased the B. pertussis bacterial burden in the respiratory tract. Bacteria were quantified by serially diluted CFU after vaccination and intranasal challenge. CFU counts were determined from lung homogenate (A), trachea (B), and nasal lavage (C) 1.5 h, 1 day, and 9 days after B. pertussis challenge. The results are shown as means ± the SEM (*n *=* *2 to 4). *P* values were determined by one-way ANOVA with Dunnett’s *post hoc* test (*, *P *<* *0.05; **, *P *<* *0.01; ***, *P *<* *0.001; ****, *P *<* *0.0001 [compared to the mock-vaccinated challenge group]).

Next, we wanted to assess whether mucosal immunization could protect against *B. pertussis* burden in the respiratory tract. The bacterial burden in the respiratory tract was determined 1 h, 1 day, and 9 days after *B. pertussis* challenge. The bacterial burden was measured at 1 h postchallenge (*n* = 2) to assess the potential bacterial loss for our original challenge dose. In the lung, trachea, and nasal lavage fluid, we measured ∼10^6^ CFU at 1 h postchallenge (Fig. 7A to C). At day 1 postchallenge, there was a significant 98.5% reduction in bacterial burden in the lungs of IM-aP-immunized rats compared to MVC controls (Fig. 7A). IM-wP-, IM-aP-, IN-aP-, and OG-aP-vaccinated rats all experienced a significant decrease in bacterial burden in the lung at day 9 postchallenge compared to MVC rats (Fig. 7A). There was also a significant decrease in bacterial burden in the trachea at both days 1 and 9 postchallenge in all vaccinated rats compared to MVC controls (Fig. 7B). At day 1 postchallenge, there was a significant 86 to 97% reduction in bacterial burden in all vaccinated rats compared to MVC rats in the nasal cavity (Fig. 7C). At day 9 postchallenge, we did not measure any significant differences between groups since most of the bacteria were cleared from the nasal cavity (Fig. 7C). Overall, we observed that IN-aP- and OG-aP-vaccinated rats showed a significant reduction in bacterial burden in the respiratory tract compared to the MVC control group at days 1 and 9 postchallenge (Fig. 7A to C).

### wP immunization induces a proinflammatory cytokine response compared to mucosally vaccinated Sprague-Dawley rats.

Previous studies have shown that both wP immunization and *B. pertussis* infection induces a proinflammatory Th1/Th17 immune response, while aP immunization promotes a more Th2 skewed response (44–50). To compare cytokine responses in the coughing rat model through vaccination and challenge to the cytokine responses observed in the mouse and baboon models, we measured cytokines in the lung and serum. In the lung at day 1 postchallenge, we measured a significant 4-fold increase in IL-17 in MVC rats compared to IM-aP- and IN-aP-vaccinated rats (see Fig. S5A). At day 9 postchallenge, IM-wP-immunized rats had a significant increase in IL-17 compared to IM-aP-vaccinated, IN-aP-vaccinated, and MVC rats (see Fig. S5A). IM-wP-vaccinated rats also had a significant increase in Th1 cytokine IL-12p70 compared to MVC rats in the lung and serum at day 9 postchallenge (see Fig. S5A and B). IM-wP-vaccinated rats had a significant increase in the Th2 cytokines IL-4 and IL-13 in the serum compared to IM-aP- and IN-aP-vaccinated rats and a significant increase in IL-4 to MVC controls at day 9 postchallenge (see Fig. S5B). IM-wP-immunized rats also had a significant increase in granulocyte/colony-stimulating factor in the serum day 9 postchallenge compared to IM-aP-, IN-aP-, and OG-aP-vaccinated rats and MVC rats. Overall, we did not observe marked changes in cytokine responses between DTaP-vaccinated rats compared to the MVC control group; however, rats immunized with OG-aP did have a slight increase in IL-17, although this increase was not significant. The observed difference in cytokines levels could be from *B. pertussis* challenge rather than vaccination. The increase in acute inflammatory score in the lungs of IM-wP-immunized rats at day 9 postchallenge could be associated with the increase in proinflammatory cytokines. Graphs showing the statistical significance between cytokines in the serum and lung are included in the supplemental data (see Fig. S6 and S7). Our data show that the response to B. pertussis in IM-wP-immunized animals is a robust cytokine response compared to both naive and aP-vaccinated rats, which is to be expected based on work that has examined the Th17 response induced by whole-cell pertussis vaccines.

*B. pertussis* infection induces an increase in circulating neutrophils in the blood, as well as white blood cells and lymphocytes (51–56). In the present study, we utilized hematology and flow cytometry to evaluate these populations. Hematology analysis revealed a significant increase in blood lymphocytes in the IN-aP-vaccinated rats compared to MVC rats postchallenge; however, no other differences in white blood cell counts in the blood were observed in the other vaccinated groups (see Fig. S8A and B). At day 1 postchallenge, there was a significant decrease in circulating neutrophils in the blood of IN-aP-immunized rats compared to MVC rats (see Fig. S8C). Flow cytometry analysis revealed minimal differences in the numbers of neutrophils and B cells at days 1 and 9 postchallenge in all groups (see Fig. S8E and F). Based upon these data, subtle differences in various circulating cell populations were observed following IN-aP vaccination.

### Serological responses correlate with bacterial clearance in the respiratory tract.

Currently, no definitive correlates of protection (CoP) for vaccines to protect against *B. pertussis* have been established, despite many years of research (22). Th17 responses, as well as Trms, correlate with strong protection in mice and baboons. Antibodies to PT/FHA/PRN do not always correlate with protection in humans. In an effort to more precisely define correlates using the rat model, we aimed to utilize the coughing phenotype and bacterial burden to identify the nature of how each vaccine protects (o.g., i.n., and i.m.; acellular or wP). Previous work in our lab performed by Wolf et al. illustrated that serum anti-*B. pertussis*, anti-FHA, and anti-PT IgG antibody titers in the serum following i.n. vaccination in mice correlated with the decrease in bacterial burden in the lung following *B. pertussis* challenge (28). Previous studies have shown that serum anti-*B. pertussis* IgG antibodies induced from wP vaccination correlate with protection against bacterial burden in the lungs of *B. pertussis*-challenged mice (57). Here, we hypothesized that antigen-specific serum IgG and mucosal IgA antibodies correlate with decreased bacterial burden and cough, since IN-aP and OG-aP vaccination induced systemic and mucosal antibody responses. To test this hypothesis, we generated correlograms to evaluate both negative and positive correlations elicited by each vaccination route. Correlograms are an analysis tool that can be used to determine whether the relationship observed between variables (i.e., bacterial burden and antibody titers) is random or not (58). If the relationship between the two variables is random the *R*^2^ correlation value is at or near zero (58). The relationship is considered correlative if the *R*^2^ value is approximately positive or negative (58). Significant positive nonzero correlation values demonstrate a positive correlation between variables, whereas significant negative nonzero values represent a negative correlation (58). Negative correlations are observed when two variables are inversely related to one another; that is, when one variable increases, the other decreases. With these data, as bacterial burden would drop, then the correlate would increase (negative correlation; inverse). A positive correlation would mean that as the bacterial burden increases, so does the correlate to which it is being compared.

**FIG 8:**
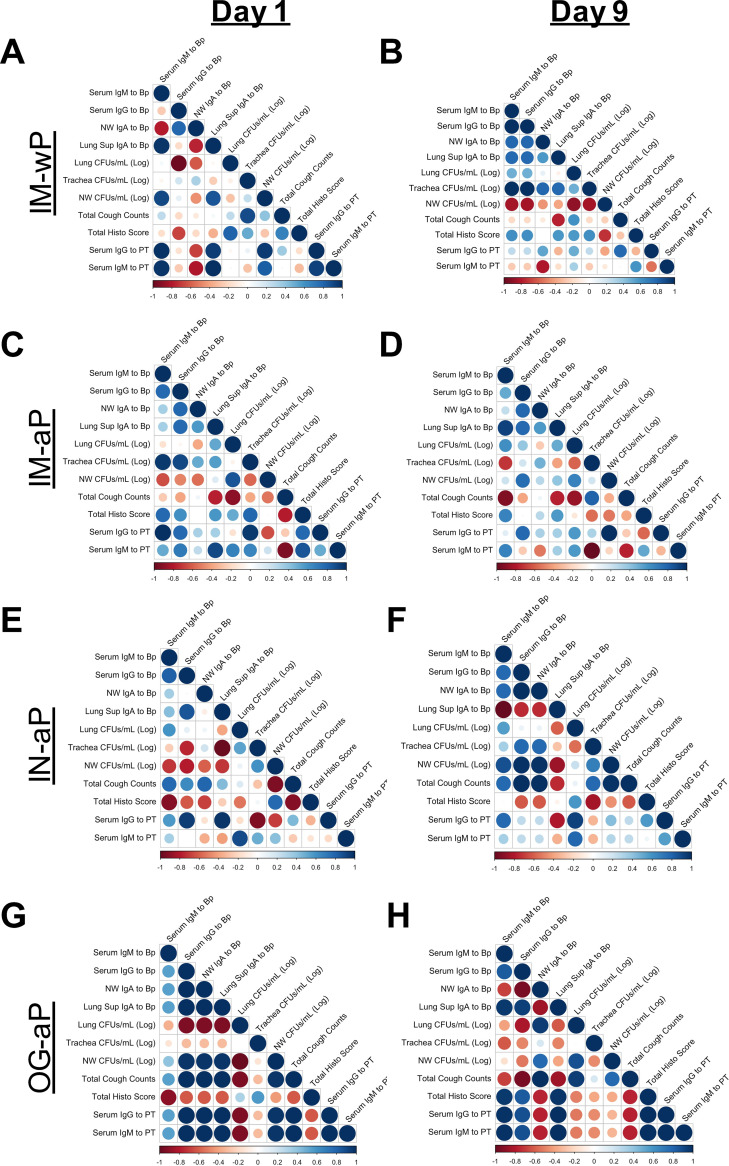
Systemic and mucosal anti-*B. pertussis* and anti-PT antibodies correlate with observed protection. Correlograms were generated using the observed data for IM-wP (A and B), IM-aP (C and D), IN-aP (E and F), and OG-aP (G and H) immunizations. Program R was used to make correlation graphs from raw data for both day 1 and day 9 postchallenge. *R*^2^ values were generated when generating the correlograms. Positive correlations are annotated by blue circles, while the negative correlations are annotated by red circles. The size of the circle indicates the strength of the correlation.

IM-wP-vaccinated rats had strong negative correlations (protective) between serum anti-*B. pertussis* IgG antibodies to both bacterial burden in the lung (*R*^2^ = −0.97) at day 1 postchallenge and the nasal cavity (*R*^2^ = −0.84) at day 9 postchallenge (Fig. 8A and B). At day 1 postchallenge, we observed negative correlations (protective) between systemic anti-*B. pertussis* IgM and anti-PT IgG antibodies in IM-aP-vaccinated rats to bacterial burden in the nasal cavity (R^2^ = −0.64 and −0.64, respectively). In addition, negative correlations were observed between anti-*B. pertussis* IgM antibodies and bacterial burden in the trachea (*R*^2^ = −0.72) at day 9 postchallenge (Fig. 8C and D). IM-aP-vaccinated rats also had negative correlations between total cough counts over the course of challenge with IgG and IgM antibodies to *B. pertussis* and PT (Fig. 8D). We expected that i.n. immunization would induce negative correlations between both serum- and mucosa-specific antibodies compared to bacterial burden in the lung, trachea, and nasal cavity at day 1 postchallenge, which is what we observed (Fig. 8E). Lung anti-*B. pertussis* IgA antibodies also negatively correlated with total cough count (*R*^2^ = −0.74) and bacterial burden (*R*^2^ = −0.6) in the lung at day 9 postchallenge for IN-aP-vaccinated rats (Fig. 8F). We observed strong negative correlations between serum IgG and mucosal IgA antibody to bacterial burden in the lungs (*R*^2^ = −0.93 and −0.93, respectively) in OG-aP-vaccinated rats at day 1 postchallenge despite the overall lower serological responses (Fig. 8G). At day 9 postchallenge, there was a negative correlation between serum anti-*B. pertussis* IgG antibodies to bacterial burden in the lungs (*R*^2^ = −0.81), tracheas (*R*^2^ = −0.49), and nasal cavities (*R*^2^ = −0.52) in OG-aP-immunized rats (Fig. 8H). We also noticed that OG-aP vaccinated rats had negative correlations between serum IgG and mucosal IgA antibodies in the lung to total cough count (*R*^2^ = −0.94 and −0.84) at day 9 postchallenge (Fig. 8H). Positive correlations (nonprotective) were observed between bacterial burden and total inflammatory score in IM-wP-, IM-aP-, and OG-aP-immunized rats (Fig. 8A, C, and G). The bacterial burden also positively correlated with total cough counts at day 9 postchallenge (Fig. 8B, D, F, and H). By utilizing correlograms, strong negative correlations between serum serological responses and bacterial burden were observed in IM-wP- and IM-aP-immunized rats. In addition, our data underscore the idea that both systemic and mucosal antibodies correlate with the observed *B. pertussis* clearance in the respiratory tract and protection from *B. pertussis*-induced cough elicited by IN-aP and OG-aP vaccination, highlighting the observed differences between vaccination routes.

## DISCUSSION

The immunity induced by aP vaccines is relatively short-lived; thus, DTaP/Tdap-vaccinated individuals are still capable of *B. pertussis* transmission (12, 59). We have recently reinvestigated the rat model of pertussis to further understand *B. pertussis* pathogenesis from current circulating *B. pertussis* strains (such as CDC isolate D420) (43). The coughing rat model of pertussis is a tool that can be used to evaluate bacterial burden in the respiratory tract and also evaluate vaccine-induced immunity against cough and respiratory function (36–41, 60). In our previous study and in the present study, we utilized i.n. challenge to infect rats in order to study immune responses, the bacterial burden, and the coughing phenotype (61). Here, we evaluated mucosal vaccination with DTaP in the coughing rat model of pertussis. To our knowledge, this study is the first to evaluate both i.n.- and o.g.-administered DTaP in the coughing rat model of pertussis. The data presented here suggest that mucosal immunization protects not only against bacterial burden but also against *B. pertussis*-induced cough, as determined by WBP (Fig. 2 and 7).

Vaccine-mediated immunity has been studied in the coughing rat model of pertussis. Utilizing audio tape recorders, Hall et al. illustrated in 1998 that three- and five-component aP vaccines administered subcutaneously could protect against *B. pertussis*-induced cough (41). In another study, wP immunization administered intraperitoneally also decreased the incidence of cough in rats (39). Neither study noted protection against bacterial colonization. Here, in the present study, WBP was used to investigate *B. pertussis*-induced cough in IN-aP- and OG-aP-vaccinated rats, as well as to measure the bacterial burden in the respiratory tract. Our data support that mucosal administration of DTaP protects against bacterial burden in the respiratory tract and reduced *B. pertussis*-induced cough (Fig. 2 and 7). In addition, IN-aP vaccination reduced bronchial constriction in the lung that is elicited by *B. pertussis* infection (Fig. 4). Previous studies evaluating wP and aP vaccines in the rat model of pertussis used one human dose per rat for immunization prior to challenge (39, 41). In an effort to best model an appropriate human-to-rat dose, we utilized a 1/5 human dose to prime and boost based on the relative sizes of rats compared to mice. We have reported that a 1/40 human dose is protective in mice, and rats are roughly 10× the weight of mice (62). One caveat of this study is that we did not evaluate other human-to-rat titrations of aP. Identification of a minimal protective rat dose would allow for the investigation of vaccine efficacy of new potential antigens/adjuvants in this model (62).

Mucosal immunization has been of particular interest in the pertussis field. We and others have recently evaluated i.n. immunization of DTaP in *B. pertussis*-challenged mice (27, 28, 63–65). Intranasally DTaP-vaccinated mice were protected against *B. pertussis* challenge and also generated both systemic and mucosal antibodies (27, 28). Live attenuated strain BPZE1 administered i.n. was protective against *B. pertussis* challenge in mice and baboons and is currently in phase 2 of clinical studies (33, 34, 66, 67). BPZE1 immunization induces both anti-*B. pertussis* IgG and IgA antibodies systemically and leads to an increase in resident memory T cells in the lung (33, 35). Oral immunization has also been investigated as a possible vaccination strategy against pertussis. Oral immunization of heat-inactivated *B. pertussis* not only protected newborns against *B. pertussis* challenge but also generated serum and saliva antibody titers (30). Recombinant technologies have led to the development of live attenuated Salmonella strains presenting *B. pertussis* antigens (68, 69). Oral immunization of the Salmonella Typhimurium *aro* vaccine strain harboring the gene for PRN resulted in reduced bacterial colonization in the lung following *B. pertussis* challenge (68). A Salmonella dublin *aroA* mutant expressing the gene for FHA was also orally administered as a vaccine in mice (69). Vaccination with this strain induced IgG and IgA antibody titers to FHA in sera and in the gut (69). The present study shows that mucosal immunization induced not only systemic anti-*B. pertussis* IgG but also anti-*B. pertussis* IgA antibodies that likely play role in clearance at the mucosa (Fig. 4, 5, and 7).

CoP is defined as the immune response that is statistically accountable for the observed protection (70). While no CoP has yet to be fully agreed upon against pertussis in humans, anti-PT IgG levels of >5 IU/ml are associated with protection in humans (71). In mice, i.n. administration of DTaP induced anti-*B. pertussis*, anti-FHA, and anti-PT IgG antibodies, while wP vaccination induced serum anti-*B. pertussis* IgG antibodies that correlated with protection against *B. pertussis* (28, 57). Here, in this study, we generated correlograms between all vaccinated groups to identify correlations between variables in the coughing rat model of pertussis, which has yet to be established (Fig. 8). Our results indicate that the bacterial burden in the lung, trachea, and nasal cavity negatively correlates with systemic anti-*B. pertussis* and -PT IgM and anti-*B. pertussis* IgG antibodies in IM-wP- and IM-aP-immunized rats, while systemic and mucosal antibodies correlate with bacterial clearance in IN-aP- and OG-aP-vaccinated rats (Fig. 8). Antibodies generated after immunization also negatively correlated with a decrease in total cough counts in vaccinated rats (Fig. 8). These results suggest that the increase in systemic and mucosal antibodies induced from i.n. and o.g. vaccinations correlates with protection against a *B. pertussis* burden in the respiratory tract and against *B. pertussis*-induced cough. It is important to note that OG-aP-immunized rats did not generate significant serum antibody titers to the whole bacterium until day 9 postchallenge (Fig. 4B). Also, two OG-aP-immunized rats had an increase in anti-PT IgG antibody titers in the serum and anti-*B. pertussis* IgA antibodies in the lung (Fig. 4D and 5A). We did, however, detect antigen-specific B cells in the bone marrow, and three of the four rats had low levels of IgA in the lungs in OG-aP-immunized rats (see Fig. S3). We hypothesize that this could be because limited amount of vaccine that successfully travels to the gut-associated lymphoid tissue (GALT) for the generation of an immune response. Oral vaccines have to travel through increased pH in the stomach, while limited absorption and availability for antigen recognition also occur in the gastrointestinal tract (72). Increases in the dose or the number of doses or changes to the delivery of the vaccine in an encased vehicle are potential methods to increase orally vaccinated immune responses. Targeting of vaccine to intestinal M cells for antigen presentation has also been shown to increase oral vaccine efficacy (73). Though we did not study new adjuvants here, we hypothesize that adjuvants can aid in stimulating a protective mucosal immune response. These approaches could all potentially increase the efficacy observed through oral vaccination of aP. Furthermore, to deliver the vaccine to the gut, one could envision novel delivery mechanisms such as gelatin-coated chewables similar to the gummy vitamins that are now popular. One caveat that should be mentioned is that we did not investigate the T-cell responses (Th1/Th2/Th17/Trm/Tem) in rats, but future studies will incorporate this into the study design. Furthermore, we did not observe the vast changes in cytokine responses in this model that have been observed in mouse and baboon models of pertussis, such as IL-6 and IL-17.

We have proposed a summary for mechanism for oral vaccination of aP (see Fig. S9). Generation of antibodies relies on both antigen presentation and antigen recognition. Based on our serological analysis, OG-aP-immunized rats developed measurable anti-*B. pertussis* IgG antibodies at day 9 postchallenge (Fig. 4B). In addition, two of the four OG-aP-immunized rats developed both anti-PT IgG and anti-*B. pertussis* IgA antibodies in the serum and lung, respectively (Fig. 4D and 5A). Detection of both IgG and IgA antibodies suggest both germinal center reactions and *B. pertussis*-specific humoral immunity. ELISpot data identified the presence *B. pertussis*-specific B cells in the bone marrow in OG-aP-vaccinated rats, and we hypothesize that these B cells home from the lymph to the marrow awaiting recall (see Fig. S3). Further studies are needed not only to confirm germinal center reactions in the Peyer’s patches but also to determine whether direct class switching and terminal differentiation occurs.

In summary, mucosal vaccination not only protected against bacterial burden in the respiratory tract of challenged rats but also protected against *B. pertussis*-induced cough and respiratory distress, as measured by WBP (Fig. 2, 3, and 7). It is critical that “next-generation pertussis vaccines” protect against bacterial colonization in the lung, nasal cavity, and trachea, since disease manifestations are dependent on bacterial colonization of the lung and trachea, mediated by FHA and fimbriae (74, 75). The coughing rat model of pertussis can be used as a tool to truly evaluate not only bacterial burden but also other clinical manifestations of pertussis, such as cough and respiratory distress, that have not been characterized in the mouse model of pertussis. Interestingly, we have recently performed pilot studies with aerosolization of *B. pertussis*, and we have confirmed that aerosol delivery can result in a similar bacterial burden at day 1 postchallenge. We expect to further investigate the aerosol challenge model with rats as well.

We observed that both i.n.- and o.g.-immunized rats generated anti-*B. pertussis*-specific IgG antibodies in the serum, while i.n. vaccination also generated significant anti-*B. pertussis* IgA antibody titers in the nasal cavity after challenge (Fig. 4 and 5). IN-aP- and OG-aP-immunized rats were protected against neutrophilic infiltration in the lung (Fig. 6). Further investigation into mononuclear involvement via i.n. vaccination is needed to delineate potential beneficial or undesired involvement in this model. Histological analysis prior to vaccination could also identify whether the observed mononuclear infiltrates are induced via i.n. vaccination solely. To fully detail the most effective route of immunization, further studies are needed to determine whether mucosal immunization addresses the problem of waning vaccine immunity. Future studies testing additional adjuvants and vaccine doses would also improve mucosal vaccine formulations. Overall, our data continue to support the potential of a mucosal vaccination against *B. pertussis.*

More studies are needed to fully characterize the immune response generated following IN-aP and OG-aP vaccination in rats, as well as vaccine-mediated immunity from vaccination in the coughing rat model of pertussis. T-cell immune responses that have been shown to play a role in natural and vaccine-mediated immunity against pertussis have yet to be evaluated in the coughing rat model of pertussis due to the limited availability of resources to adequately measure T-cell responses. Vaccine-mediated memory has yet to be evaluated in the coughing rat model of pertussis. Additional research is needed to critically assess vaccine mediated memory, since it is essential that the next generation of pertussis vaccines induces longer-lasting memory than do current vaccines. Future work is also needed to evaluate mucosal immunization against current circulating strains of *B. pertussis* since current strains are genetically divergent from strains of the past, with the goal of making the most efficacious vaccine against *B. pertussis*.

## MATERIALS AND METHODS

### Vaccine composition and administration.

INFANRIX (GSK, catalog no. 58160-810-11) acellular pertussis human vaccine (DTaP) and the National Institute for Biological Standards and Control WHO whole-cell pertussis vaccine (NIBSC code 94/532) were used for this study. Vaccines were diluted with endotoxin-free Dulbecco PBS (Thermo Fisher Scientific, catalog no. TMS012A) to a concentration of 1/5 the human dose. Vaccines were diluted and administered no more than 1 h from composition. The first dose of vaccine was administered to 3-week-old (50 g) female Sprague-Dawley rats (Charles River, catalog no. 001CD). When the animals were 6 weeks old, a booster vaccine of the same dose was administered, followed by *B. pertussis* challenge at 8 weeks of age. Intramuscular (i.m.)-vaccinated rats received 100 μl in the right thigh muscle of the hind limb. Intranasal (i.n.)-immunized rats were first anesthetized with isoflurane until breathing was minimal. Rats then received 50 μl of vaccine in each nostril for a 100-μl dose. Oral gavage (o.g.)-vaccinated rats received a 100-μl dose delivered via a curved 18-gauge feeding needle (Fisher Scientific, catalog no. NC9349775). The MVC control group received 100 μl of the same endotoxin-free PBS used to dilute the vaccines in the right thigh muscles of the hind limb. At 1 week postprime, 2 weeks postprime, and 1 week postboost, blood was collected via saphenous blood draws for serological analysis. Then, 5-mm animal lancets (Fisher Scientific, catalog no. NC9891620) were used for the blood draw. Blood was collected in capillary tubes (Fisher Scientific, catalog no. NC9059691) for centrifugation. Blood was spun at 15,000 × *g* for 3 min, and serum samples were collected and stored at −80°C until analysis.

### *Bordetella pertussis* strains and growth conditions.

*B. pertussis* strain D420 (GenBank accession no. LN849008.1) was cultured on Bordet-Gengou (BG) agar (Remel, catalog no. R45232) supplemented with 15% defibrinated sheep blood (Hemostat Laboratories, catalog no. DSB500) (1). Bacteria cultured in BG plates were incubated for 48 h at 36°C. Using polyester swabs (Puritan, catalog no. 22-029-574), we transferred *B. pertussis* into 20 ml of Stainer-Scholte liquid media (SSM) in new 125-ml flasks (Thermo Fisher Scientific, catalog no. FB500125) (76). Bacterial cultures were allowed to grow at 36^°^C for 24 h inside a shaking incubator at 180 rpm.

### Intranasal challenge.

Vaccinated 8-week-old ∼200-g female Sprague-Dawley rats were then challenged. *B. pertussis* was grown as illustrated above. Rats were anesthetized with ketamine and xylazine at 50 to 100 and 5 to 10 mg/kg, respectively, and challenged with 10^8^ CFU in 100 μl i.n., 50 μl in each nostril. The body weight of each rat was recorded before bacterial challenge, and body weights were taken posteuthanasia to calculate the percent weight change. At days 1 and 9 postchallenge, the rats were euthanized. Upon euthanasia, blood was collected via cardiac puncture and transferred into EDTA (BD, catalog no. 365974) and serum separation (BD, catalog no. 026897) tubes. After cardiac puncture, 250 μl of blood was collected into EDTA tubes for flow cytometry and ProCyte (IDEXX) hematology analysis, while the remaining blood was collected in serum separation tubes to isolate the serum via centrifugation (15,000 × *g* for 3 min) and then used for serological and cytokine analysis. To determine the bacterial burden in the respiratory tract, the lungs and tracheas were excised separately and homogenized. Lung weights were recorded following excision before homogenization. The lungs were then collected in gentleMACS C-tubes (Miltenyi Biotec, catalog no. 130-096-334) in 2 ml of PBS and homogenized using a Miltenyi Biotec tissue dissociator (catalog no. 130-095-927). A polytron homogenizer was used to homogenize the trachea in 1 ml of PBS. The bacterial burden in the nares was determined by flushing 2 ml of sterile 1× PBS through the nares, and samples were collected for serial dilution and plating. Serial dilutions of the homogenates and nasal collection were plated on BG plates supplemented with ceftibuten (Sigma-Aldrich, catalog no. SML0037) at 10 μg/ml.

### Serological analysis.

ELISAs were performed to measure the antibody titers of vaccinated and infected rats. *B. pertussis*-specific whole-bacterium ELISA plates were coated with 50 μl of 10^8^ CFU of *B. pertussis* grown as described above for infection. Antigen-specific antibody titers to PT (List Biological Laboratories, catalog no. 180) were measured by coating ELISA plates with 50 μl of antigen per well. Antigen-coated plates incubated overnight at 4°C. After incubation, the plates were washed with 1× PBS–Tween 20 and blocked with 5% skimmed milk for 2 h at 37°C. After blocking, ELISA plates were washed, and serum samples from the saphenous blood draws and blood collected from cardiac puncture posteuthanasia were serially diluted down the ELISA plate, followed by incubation for 2 h at 37°C. To measure respiratory IgA antibody titers in the lungs and nasal lavages, lung homogenate supernatant and nasal lavage were added, followed by incubation for 2 h at 37°C. After incubation, the ELISA plates were washed as described above, and 100 μl of secondary goat anti-rat IgG (Southern Biotech, catalog no. 3030-04), goat anti-rat IgM (Southern Biotech, catalog no. 3020-04), or goat anti-rat IgA (MyBioSource, catalog no. MBS539212) was added to the plates at a dilution of 1:2,000 in PBS–5% milk, followed by incubation for 1 h at 37°C. The plates were then washed again, 100 μl of *p*-nitrophenyl phosphate substate (Thermo Scientific, catalog no. 37620) was added, and the plate was developed for 30 min at room temperature. After development, the colorimetric signal of the ELISA plate at *A*_450_ was measured by using a BioTek Synergy H1 microplate reader. Antibody titers were considered positive if values were higher than the baseline value. The baseline value for each sample was set as double the average value of the blank, to which no serum, lung supernatant, or nasal lavage had been added. The limit of detection was set at 50, and any samples with a titer value less than that were set to 50.

### ELISpot assay.

An ELISpot assay (ImmunoSpot, catalog no. mTgG-SCE-1M/2) was used to analyze antigen-specific B cells in the bone marrow. The right hind femur of each rat was removed, placed in Dulbecco modified Eagle medium, and frozen at −80°C until analysis. The bones were then thawed in a water bath at 37°C, immediately transferred to spin tubes, and spun at 1,000 × *g* for 3 min to collect the bone marrow. The bone marrow was passed through a 70-μm filter to create a single-cell suspension. Cells were centrifuged at 350 × *g* for 5 min, and the cell pellet was resuspended in CTL test B media (ImmunoSpot). D420 was cultured as described above and coated the 96-well ELISpot plate, as described above for the ELISA. The plate was incubated overnight at 4°C and then washed with 1× PBS before cells were added. Three serial dilutions of cells (1.25 × 10^6^, 3.13 × 10^5^, and 1.56 × 10^5^) were added per well, followed by incubation at 37°C overnight. Rabbit anti-rat IgG antibody (Abcam, catalog no. ab6733) was used to replace the anti-murine IgG detection antibody that was provided with the kit. The rest of the protocol was followed according to the manufacturer’s instructions. The ELISpot plates were imaged and analyzed using an ImmunoSpot S6 Entry analyzer and CTL software.

### Analysis of cough and bronchiole restriction using WBP.

Buxco FinePointe WBP (DSI) was used to quantify respiratory function during infection. Every day following *B. pertussis* challenge and 1 day before challenge (5:00 p.m.), rat respiratory profiles and coughs were measured. FinePointe software was used to record respiratory parameters. A 5-min acclimation period was used before measuring the coughs and other respiratory parameters. After acclimation, the respiratory profile was recorded for 15 min for each rat. Coughs were counted over 15 min. The enhanced pause (PenH) was calculated as follows by measuring the peak expiratory height (PEF) and peak inspiratory height (PIF) and the time it takes for the rat to breathe each breath: PenH = (PEF/PIF) × [(Te/Tr) – 1], which represents bronchiole restriction during breathing. An increase in PenH indicates an increase in pulmonary distress. Coughs were counted based on box flow changes of the subject with classical cough-like waveforms. Patented fuzzy logic criteria was used to detect and count coughs (77). Each cough in a multicough event was counted individually. The frequency (F), tidal volume (TVb), pause (PAU), minute volume (MVb), inspiratory time (Ti), and expiratory time (Te) were also recorded and analyzed over the course of infection.

### Histological assessment of the lung.

The left lobe of the lung was used for histological assessment. After excision of the left lobe, the sectioned portion was fixed in 10% formalin 48 h at 26°C. After fixation, samples were embedded in paraffin and stained with H&E by the WVU Pathology Department. Stained samples were used to characterize and score inflammation of the lung. All scorings were done by a board-certified pathologist (iHisto). Individual scores were determined using a standard qualitative scoring criterion: 0, none; 1, minimal (rare); 2, mild (slight); 3, moderate; 4, marked; and 5, severe. The presence of neutrophils in the parenchyma, blood vessels, and airway was used to score acute inflammation, and chronic inflammation was characterized by mononuclear infiltrates of the parenchyma, blood vessels, and airway. All examination and scoring were done with no knowledge of the groups.

### ProCyte analysis of blood.

Blood from the EDTA tubes was used to analyze white blood cell, neutrophil, and lymphocyte counts. Samples (25 to 50 μl) of blood were drawn from the EDTA tubes and analyzed by the ProCyte. After ProCyte analysis, the rest of the blood was used for flow cytometry analysis.

### Flow cytometry analysis.

Blood samples were lysed with 1× PharmLyse buffer (BD Biosciences, catalog no. 555899) for 20 min at room temperature. Blood samples were vortexed periodically during the 20-min incubation. After lysis, the cells were resuspended in RPMI–10% FBS to neutralize the lysis buffer and centrifuged five times at 1,000 × *g*. The cells were then washed with RPMI–10% FBS again. The cells were next resuspended in 1% FBS–PBS–5 mM EDTA. Blood samples were blocked with anti-CD32 (BD Pharmingen, catalog no. 550270) antibody for 15 min at 4°C. After blocking, the cells were labeled with CD45-Alexa Fluor 700 (BioLegend, catalog no. 202218), CD161-APC (BioLegend, catalog no. 205606), CD45R-PE-Cy7 (eBioscience, catalog no. 25-0460-82), His48-FITC (eBioscience, catalog no. 11-0570-82), CD43-PE (BioLegend, catalog no. 202812), or CD3-VioGreen (Miltenyi Biotec, catalog no. 130-119-125) (78). The samples were then incubated for 1 h at 4°C in the dark. To prepare the lung samples for flow cytometry, the lung homogenate was filtered through a 70-μm cell strainer (BioDesign Cell MicroSives, catalog no. N70R). The suspension was centrifuged at 1,000 × *g* for 5 min. The pellet was resuspended in PharmLyse buffer, followed by incubation at 37°C for 2 min. After incubation, the cells were centrifuged at 1,000 × *g* for 5 min, the lysis buffer was removed, and the cells were blocked and labeled with antibody, as described for blood samples. Both blood and lung samples were centrifuged at 1,000 × *g* for 5 min, and the pellets were resuspended in 0.4% paraformaldehyde and stored overnight at 4°C. The samples were washed with 1× PBS–5mM EDTA–1% FBS and resuspended in 1× PBS–5 mM EDTA–1% FBS for analysis. Cell samples were analyzed on a LSR Fortessa, and samples were gated and analyzed using FlowJo v10.

### Cytokine analysis.

Lung homogenates were centrifuged at 19,000 × *g* for 4 min, and the resulting supernatant was removed and stored at −80°C until further analysis. Lung supernatants and serum cytokines were measured using a ProcartaPlex multiplex immunoassay kit (Complete 14-Plex Rat ProcartaPlex panel; Thermo Fisher Scientific, catalog no. EPX140-30120-901) according to the manufacturer’s instructions. Cytokines with bead counts of <35 were invalidated.

### Generation of correlograms.

Correlograms were created using R Studio software (version 1.4.1106). Pearson correlation coefficients were calculated between each set of variables listed in the master table and then illustrated in a representative plot for each vaccine route and time point.

### Statistical analysis.

GraphPad Prism 7 was used to analyze the data. The minimum biological replicates for the challenge studies were three for the MVC control group and four rats per vaccinated group. For statistical comparisons between vaccinated groups and the MVC control group over the entire course of the infection, two-way analysis of variance (ANOVA) was used with Dunnett’s *post hoc* test. One-way ANOVA was used for comparison between vaccinated groups and MVC controls for an individual day or time point with Dunnett’s *post hoc* test. A Kruskal-Wallis test with Dunnett’s *post hoc* test was used for comparisons between groups for mucosal IgA comparisons. A ROUT test was used to identify any potential outliers during cytokine analysis of the lung.

### Ethics statement.

This challenge studies were performed in accordance with West Virginia University Institutional Animal Care and Use Committee (IACUC) protocol 1811019148.6. All research was approved by West Virginia University Institutional Biosafety Committee protocol 17-11-01.

### Data availability.

Data requests for figures provided can be addressed to the corresponding author.
