# Supplementary material for: Mucosal Immunization with DTaP Confers Protection against Bordetella pertussis Infection and Cough in Sprague-Dawley Rats
Source: Infect Immun. 2021 Nov 16;89(12):e00346-21. doi: 10.1128/IAI.00346-21 (PMC8594602; doi:10.1128/IAI.00346-21)
Supplement: Supplemental file 1 — Supplemental material. Download iai.00346-21-s0001.pdf, PDF file, 1.0 MB [file iai.00346-21-s0001.pdf]

Supplemental Figures

**FIG S1**

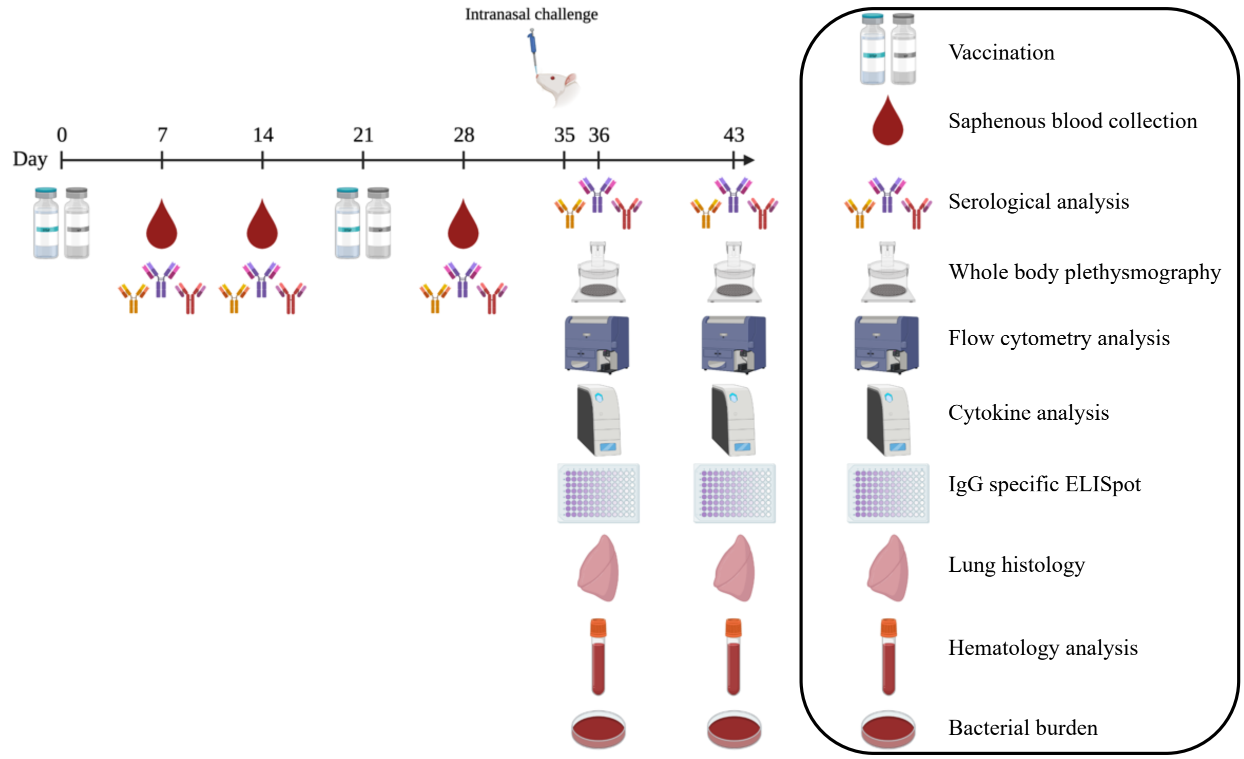


**Figure S1** Experimental design of vaccination and challenge. Three-week old Sprague Dawley rats were vaccinated IM-wP, IM-aP, IN-aP, and OG-aP 1/5$^{th}$ human dose. 21 days post prime, rats were boosted with the same corresponding vaccine. At days 7, 14, and 28 post prime, blood was collected via saphenous blood draw for serological analysis. At day 35, rats vaccinated rats were intranasally challenged with $10^8$ viable *Bp*. Cough and respiratory distress was measured using whole body plethysmography for 8 days post challenge. Antibody titers in the serum, lung, and nasal cavity was measured at days 1 and 9 post challenge. Flow cytometry was used to measure neutrophil and b cell recruitment in the lung and blood at days 1 and 9 post challenge. The left lobe of the lung was sectioned and stained with H&E for analysis of inflammation at days 1 and 9 post challenge. Antigen specific IgG ELISpot assay was performed to measure antigen specific B cells in the bone marrow at days 1 and 9 post challenge. Cytokines in the lung and serum were

measured post challenge. Hematology was used to measure blood cell populations post challenge. Bacterial burden in the respiratory tract was measured at days 1 and 9 post challenge. Created by Biorender.com.

FIG S2

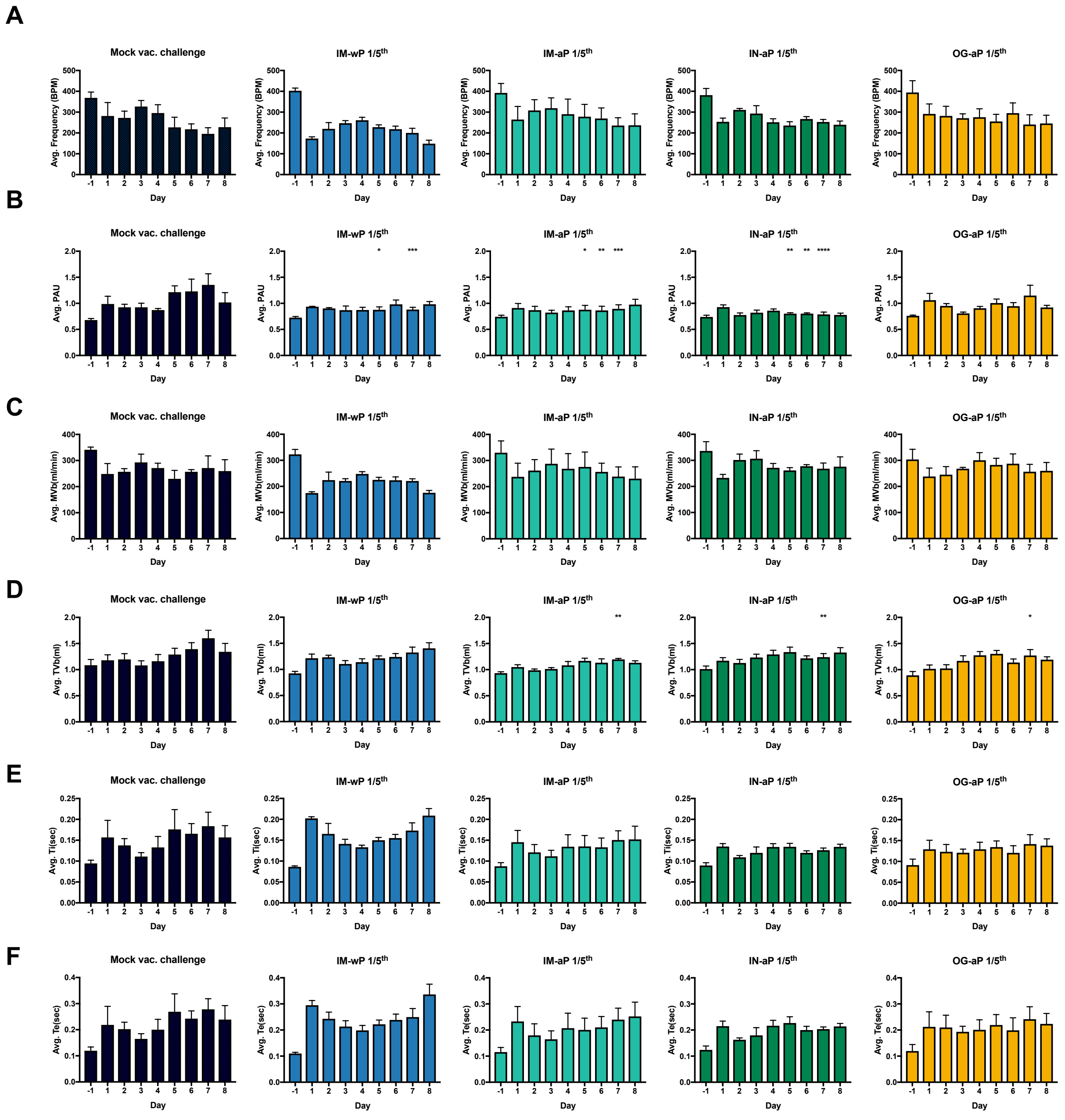


**Figure S2:** Respiratory profile of Sprague-Dawley rats vaccinated and challenged with *B. pertussis*. Every day after 5p.m. whole body plethysmography was used to analyze the respiratory capacity of immunized and challenge rats. Measurements assessed were: (A) frequency of breathes, (B) pause (PAU), (C) minute volume (MVb), (D) tidal volume (TVb), (E) time of

inspiration (Ti), and (F) time of expiration (Te). Results shown as mean ± SEM ($n = 3$-4). $P$ values were determined by two-way ANOVA with Dunnett's post hoc test, *$P < 0.05$, **$P < 0.01$, ***$P<0.001$, and ****$P < 0.0001$ compared to mock vac. challenge group.

FIG S3

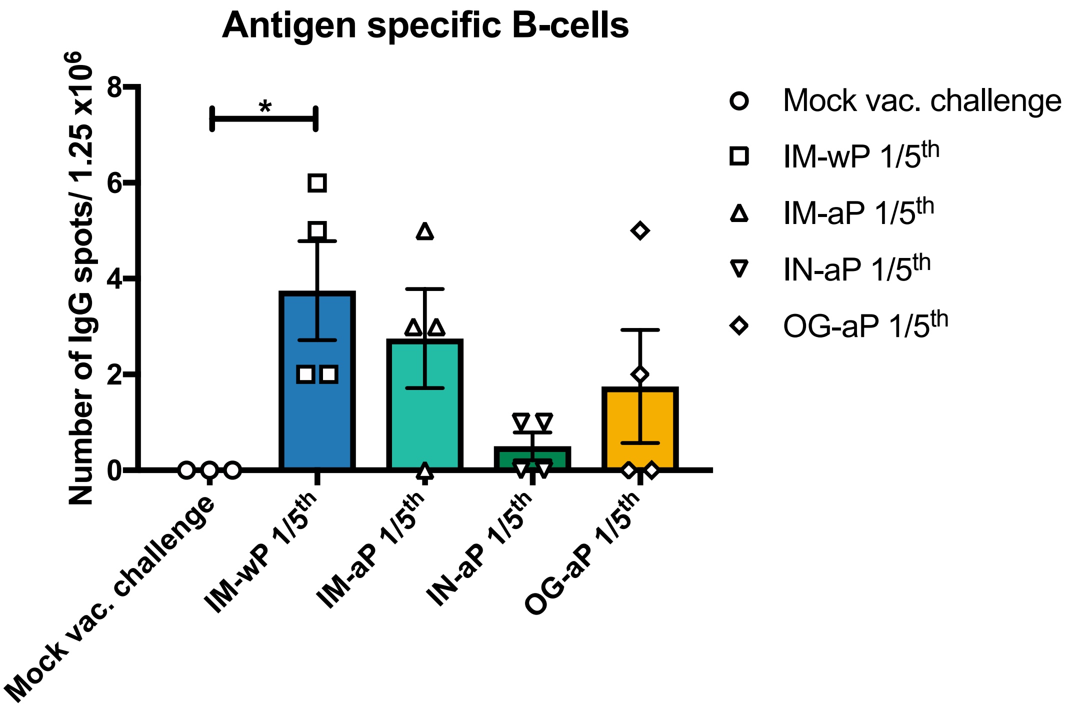


**Figure S3** ELISpot assay was performed to measure the number of *Bp* specific IgG cells from the bone marrow at day 9 post challenge. Results are shown as mean ± SEM ($n = 3$-4). $P$ values were determined by one-way ANOVA with Dunnett's post hoc test, $^{*}P < 0.05$ compared to mock vaccinated challenge group.

FIG S4

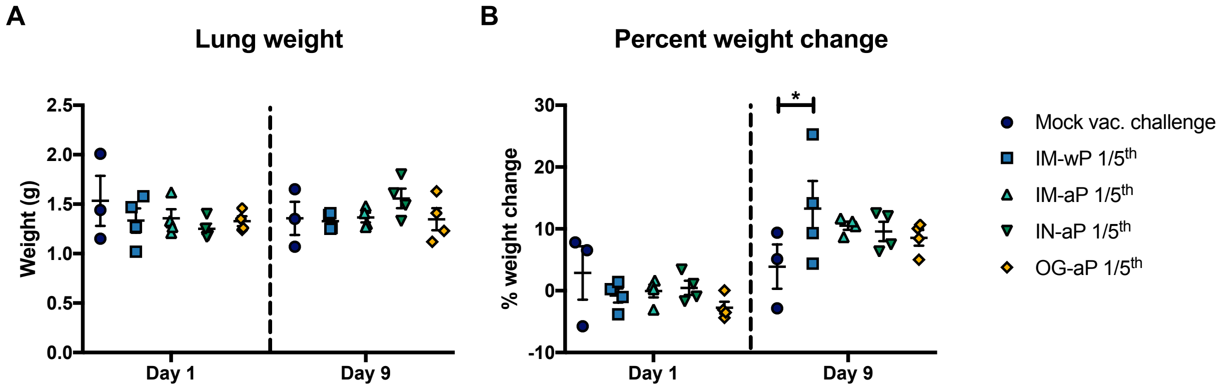


**Figure S4** Measurement of lung and body weight at days 1 and 9 post challenge. (A) Lung weight as measured immediately after euthanasia before being homogenized. Body weight for each rat was measured before challenge and immediately post euthanasia. (B) Percent weight change was calculated by taking the differences between starting weight and end weight and dividing by the initial weight multiplying by 100. Results shown as mean ± SEM ($n = 3\text{-}4$). $P$ values were determined by two-way ANOVA with Dunnett's post hoc test, $^*P < 0.05$ compared to the mock vaccinated challenge group.

**FIG S5**

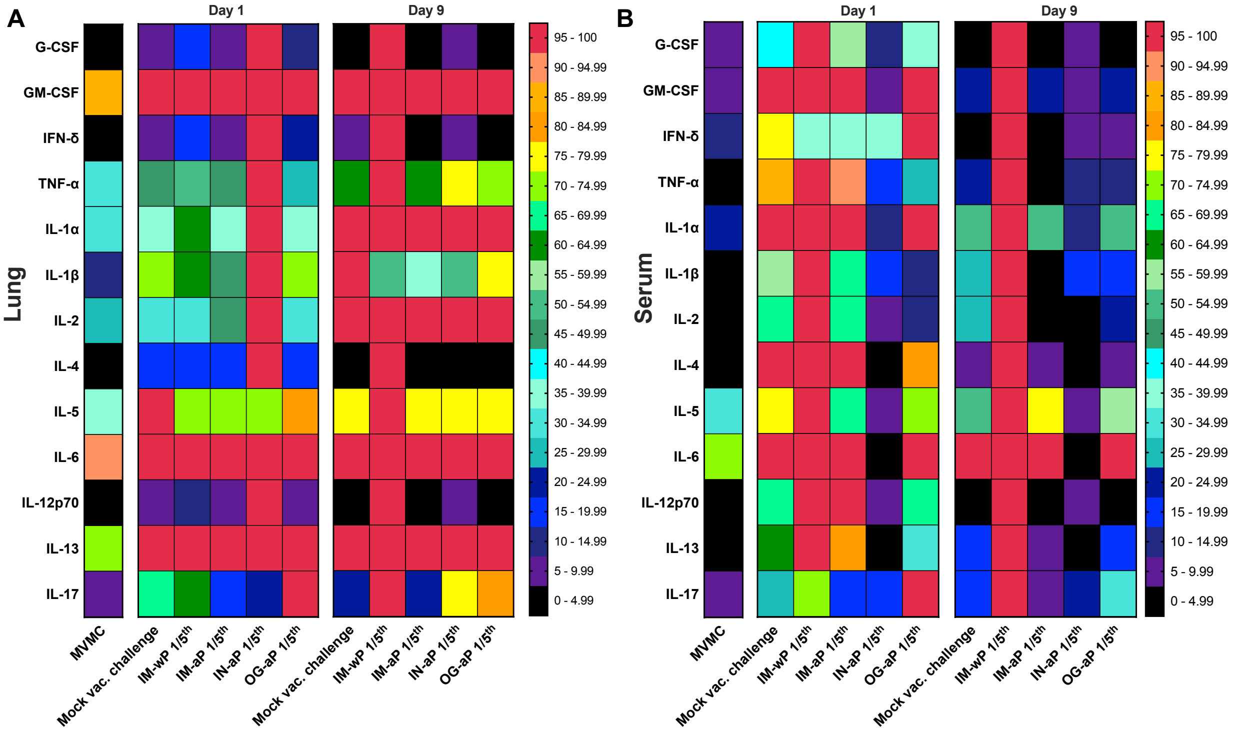


**Figure S5** Measurement of cytokines in the lung and serum at days 1 and 9 post infection. Heat map of the average percent cytokines normalized to the max cytokine measured in the (A) lung and (B) serum. MVMC (mock vaccinated mock challenge) cytokines are from rats in (Hall et al 2021). All statistical analysis comparing average cytokine values are in Fig S5-6.

## FIG S6

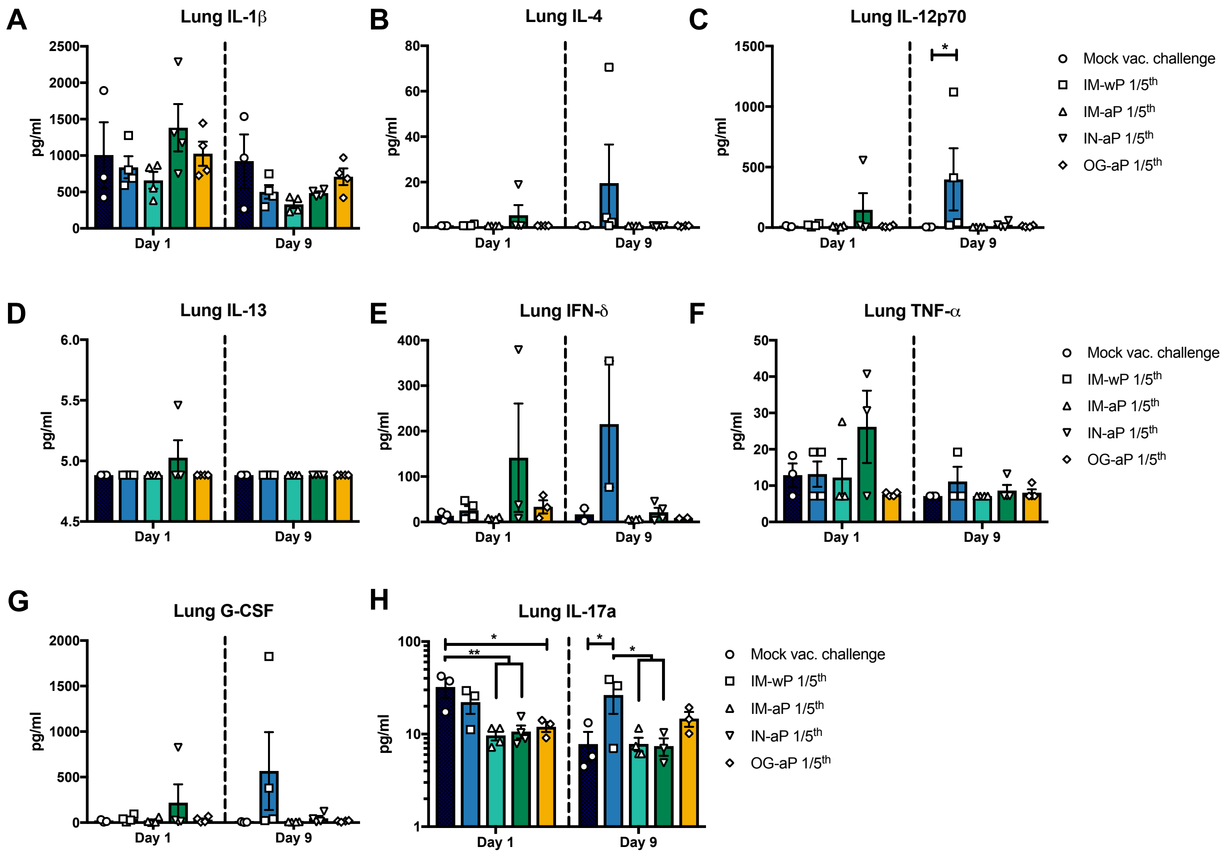


**Figure S6** Measurement of cytokines in the lung at days 1 and 9 post infection. Cytokines in the lung supernatant were analyzed using ProcartaPlex multiplex immunoassay kit. Results shown as mean ± SEM (*n* = 3-4). *P* values were determined by two-way ANOVA with Dunnett's post hoc test, $^{*}P < 0.05$, $^{**}P < 0.01$ compared between mock vaccinated challenge and vaccinated challenge groups.

FIG S7

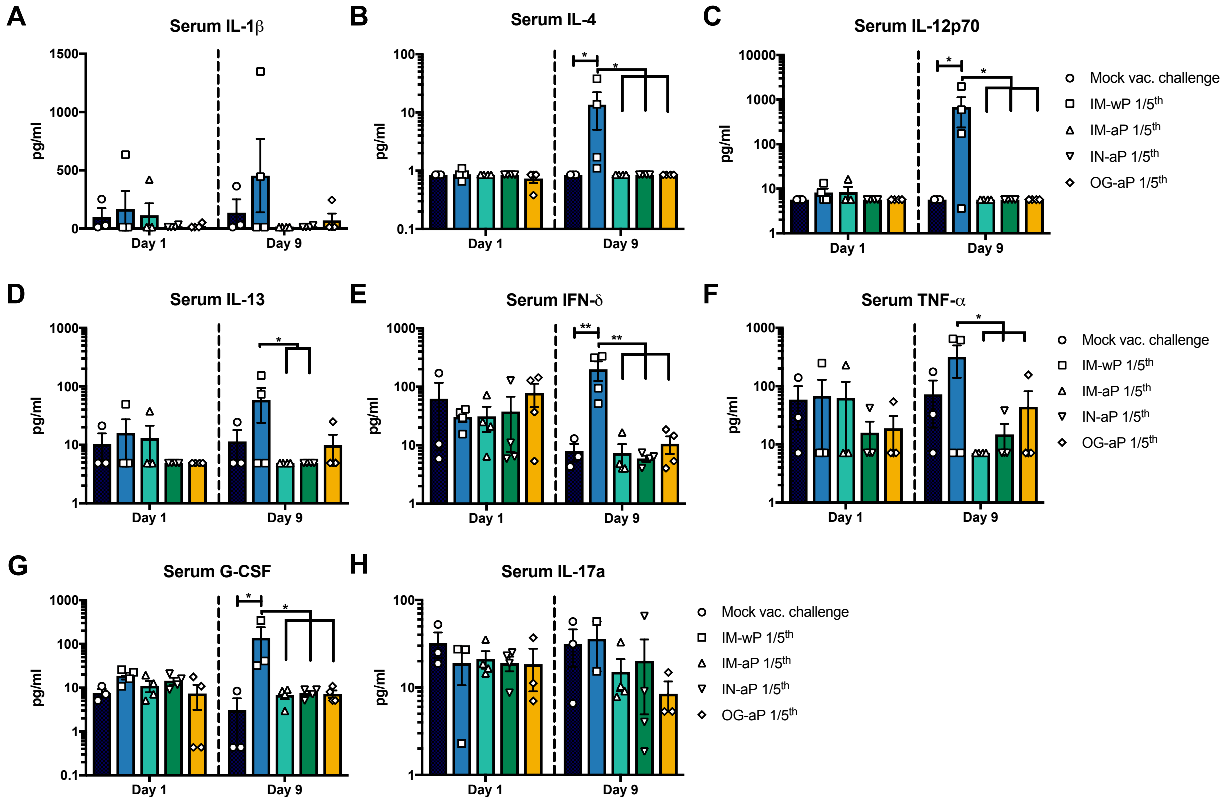


**Figure S7** Measurement of cytokines in the serum at days 1 and 9 post infection. Cytokines in the serum and lung supernatant were analyzed using ProcartaPlex multiplex immunoassay kit. Results shown as mean ± SEM ($n = 3$-4). $P$ values were determined by two-way ANOVA with Dunnett's post hoc test, $^{*}P < 0.05$, $^{**}P < 0.01$ compared between mock vaccinated challenge and vaccinated challenge groups.

**FIG S8**

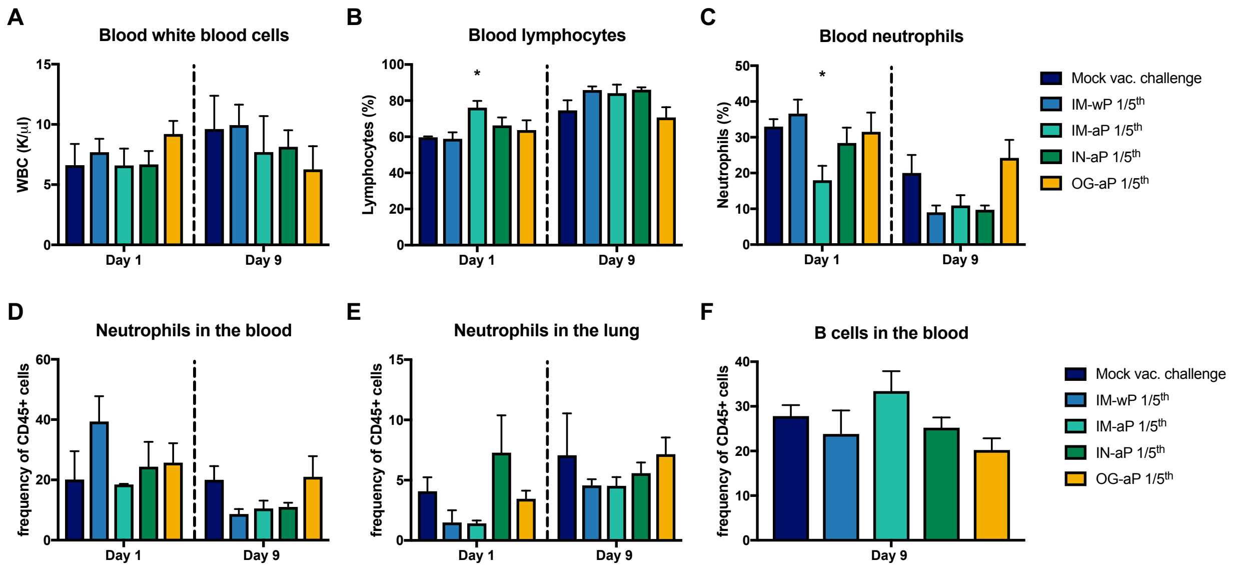


**Figure S8** Characterizing circulating cell populations in the blood and lung post infection. ProCyte hematology analyzer was used to assess (A) total white blood cells, (B) percent lymphocytes and (C) percent neutrophils in the blood post challenge. Flowcytometry analysis of circulating B cells and neutrophils in the lung and blood. (D&E) Neutrophils were gated by ($CD45^{+}$ $CD161^{-}$ $B220^{-}$ $CD43^{+}$ $His48^{hi}$), while (F) B cells were gate by ($CD45^{+}$ $CD45R^{+}$). Neutrophil and B cell quantification represented as percentage of single, CD45+ cells. Results are shown as mean ± SEM ($n$ = 3-4). $P$ values were determined by two-way ANOVA followed by Dunnett's comparison test, $^{*}P < 0.05$, compared between mock vaccinated challenge group.

**FIG S9**

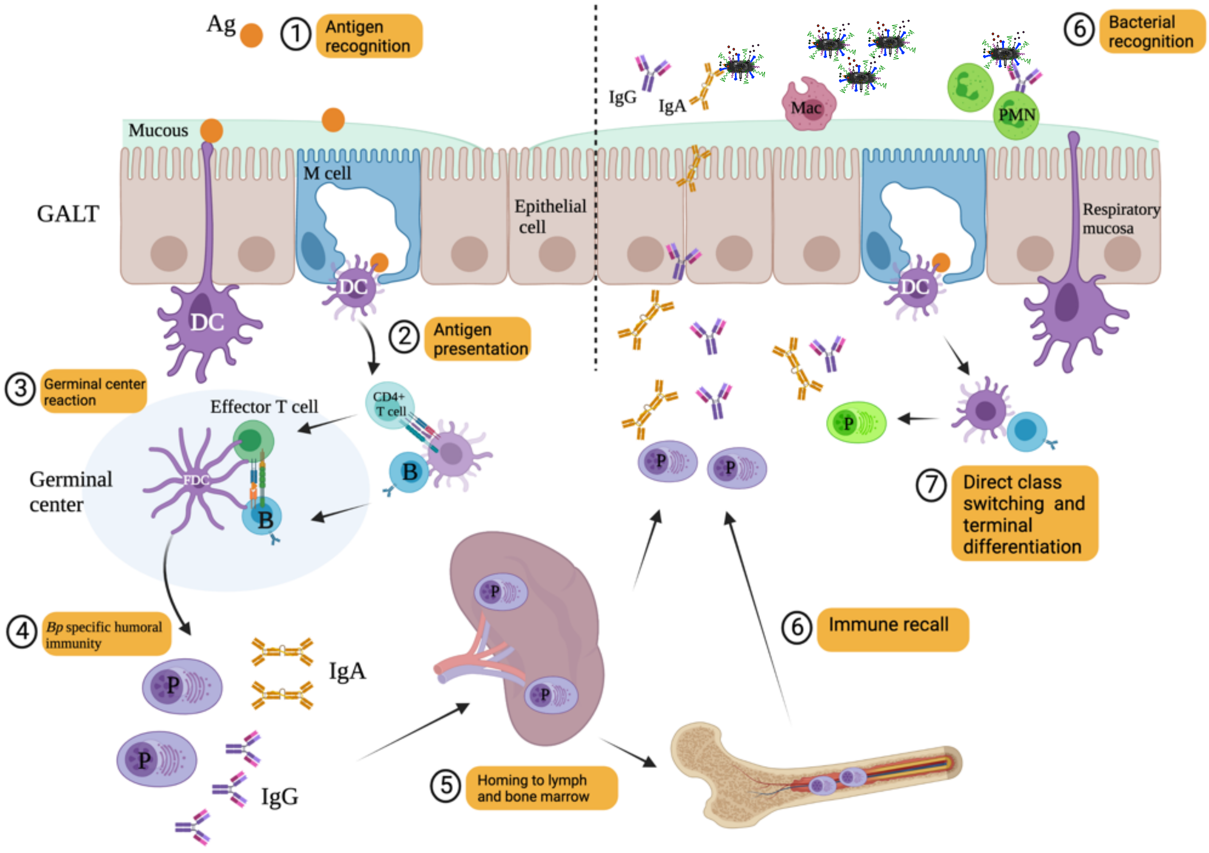


**Figure S9** Proposed mucosal immune response induced through oral vaccination. Upon oral vaccination of DTaP, antigens travel to the gut associated lymphoid tissue. Here, antigens are endocytosed by M cells or phagocytosed by DCs in the epithelial layer and antigens are presented to T and B cells in the lamina propria. Ensuing germinal center reaction occur in lymphoid follicles and induce *Bp* specific plasma cells capable of producing IgG and IgA antibodies. Generated plasma cells can home to secondary lymphoid tissue and travel to the bone marrow awaiting recall. Upon *Bp* challenge, plasma cells are recalled and migrate to the respiratory mucosa secreting IgG and IgA antibodies. Here antibodies can opsonize the bacteria and potentially play a role in antibody mediated phagocytosis of the bacteria with recruited neutrophils and macrophages. Other

potential mechanism for the generation of antibody secreting plasma cells involves direct class switching and terminal differentiation.
